# Metal Complexes in the Synthesis of Biodegradable Polymers: Achievements and Prospects

**DOI:** 10.3390/ma16206682

**Published:** 2023-10-13

**Authors:** Badma N. Mankaev, Sergey S. Karlov

**Affiliations:** 1 Chemistry Department, Lomonosov Moscow State University, 119991 Moscow, Russia; mankaev@org.chem.msu.ru; 2 N.D. Zelinsky Institute of Organic Chemistry, Russian Academy of Science, 119991 Moscow, Russia

**Keywords:** biodegradable polymer, ring-opening polymerization, main-group metal, catalysis

## Abstract

This review describes recent advances in the synthesis of homopolymers of lactide and related cyclic esters via ring-opening polymerization (ROP) in the presence of metal complexes based on group 1, 2, 4, 12, 13 and 14 metals. Particular attention is paid to the influence of the initiator structure on the properties of the obtaining homo- and copolymers. Also, a separate chapter is devoted to the study of metal complexes in the synthesis of copolymers of lactide and lactones. This review highlights the efforts made over the last ten years or so, and shows how main-group metals have received increasing attention in the field of the polymerization of lactide and related cyclic esters.

## 1. Introduction

Recently, the field of “green chemistry” has been developing very actively. Its interest was caused by current economic and environmental problems. The current concern is primarily related to the depletion of fossil resources that are used to produce traditional polyolefin “plastics”, as well as environmental problems, and in particular with the accumulation of these almost non-degradable plastics in the oceans and in the Earth’s soil. Accordingly, biodegradable polymers are designed to solve some of these problems. It is important that most of these polymers, such as polylactide, have similar physical and mechanical properties to “classic” plastics, which allow them to be processed in standard equipment.

Two classes of biodegradable polymers are usually distinguished: natural polymers—such as polysaccharides, polypeptide, polhydroxyalkanoates, etc.—and synthetic polymers, among which aliphatic polyesters, such as polylactide (PLA) and polycaprolactone (PCL), attract special attention due to their relative synthetic availability and properties, which make them suitable for the manufacturing of products.

Physical properties, hydrolysis behavior and the biodegradation of PLA can be controlled not only by changing the average molecular weight, but also its distribution. Since the lactide monomer can exist in various enantiomeric forms (L-lactide, D-lactide, *meso*-lactide), it is possible to control the stereochemistry of the polymer, which depends on the ratio of enantiomers in the starting material and the stereoselectivity during polymer chain growth, which ensures the synthesis of universal families of homopolymers with different microstructures, which have a significant influence on the physical and mechanical properties of PLA.

Upon the polymerization of D- or L-lactide, optically pure poly(D-lactide) ((RRRR)_n_) and poly(L-lactide) ((SSSS)_n_) are obtained as isotactic polymers, respectively. The polymerization of *meso*-lactide gives syndiotactic PLA ((RSRS)_n_). Poly(D-, L-lactide) can be atactic, with a random distribution of lactide configurations, or heterotactic, containing alternating D- and L-lactide units ((RRSS)_n_). The sequential copolymerization of D- and L-lactide generates polymer stereoblocks [1].

Optically pure isotactic PLA is semi-crystalline, hard and rather brittle, and has a melting point of 207 °C, but due to impurities, slight racemization and crystal defects, the typical melting point ranges from 170 to 180 °C. A 1:1 mixture of D-PLA and L-PLA has a higher melting point (T_m_ 230 °C) [2]. However, syndiotactic, heterotactic and atactic PLA are amorphous, transparent polymers with a softening point of about 60 °C [3]. The amorphous polymer is soluble in most organic solvents such as acetone, THF, benzene, acetonitrile, dioxane and chlorine solvents, while the crystalline material is soluble only in chlorine solvents or benzene when heated. From a mechanistic point of view, stereoselectivity in *rac*-LA polymerization can be achieved in two ways: namely, due to the site control mechanism (SCM), in which the metal complex has a chiral environment and can consistently distinguish L-LA from D-LA during polymerization, as well as the chain-end control mechanism (CEM), in which the metal complex and the ligand are achiral. In this case, the polymerization initiation reaction occurs without the enantiomeric differentiation of *rac*-LA, and chirality is included at the end of the growing chain. Next, the chain grows due to the approach of the monomer to the growing chain with the same chiral sense. However, in the case of the CEM, it is possible to include a monomer that does not match in chirality and further the growth with a different chirality. Thus, polymerization via the CEM in the limiting case results in two homochiral polymers, or results in the formation of a multiblock stereocopolymer (PLLA–PDLA)_n_ due to the inversion of the chiral sense during polymer chain growth [4,5].

**Figure 1:**
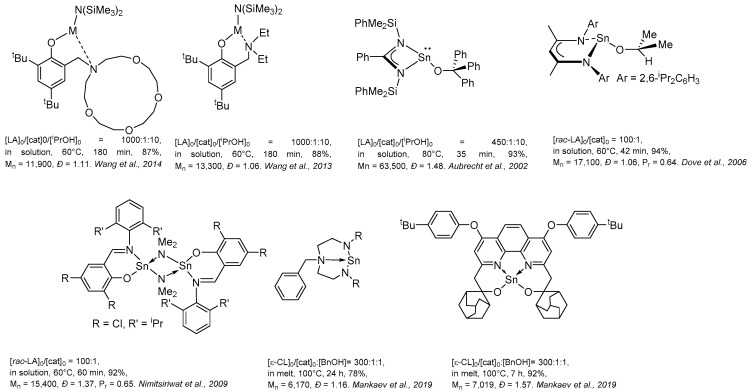
Tin initiators for ROP [12,13,14,15,16,17,18].

At present, it is used in industry as an initiator of the ring-opening polymerization of bis(2-ethylhexanoate)-tin (+2) [1,6,7,8,9]. Other tin derivatives in the oxidation state (+2) have also been studied, but there are much fewer studies of this kind than there are works devoted to complexes based on other metals (Mg, Zn, Al) [10,11]. This may be due to the expected toxicity of the resulting samples, since it is quite difficult to remove the initiator residues from the polymer. At the same time, there are no reliable data on the toxicity of tin derivatives that do not contain Sn–Alk bonds in the literature. It can be assumed that the low content of tin in the resulting polymer will not have a noticeable effect on its biocompatibility. Among the stannylenes studied as initiators of the polymerization of lactide, such as ε-caprolactone, as well as the copolymerization of lactide and glycolide, and lactide and trimethylene carbonate, there were derivatives containing the following structural fragments, in which the tin atom (+2) is di-, tri-, or tetracoordinated. The results of the polymerization experiments performed demonstrate the promise of using such complexes in catalysis (Figure 1) [12,13,14,15,16,17,18].

Narrow molecular weight distribution due to the absence of side reactions from transesterification remains relevant, given the fact that the scope of biodegradable polymers is their use in medicine as materials for controlled drug delivery systems, slow-release medication administration, tissue engineering scaffolds, bone tissue engineering, the formation of artificial organs, nerve regeneration and wound healing, long-term implants [19,20], and the synthesis of new initiators based on non-toxic metals, which produce PLA and PCL in a controlled manner with high molecular weights. Another important task in ROP is the search for new initiators that are active in the synthesis of copolymers, and allow for copolymers to be obtained that have a statistical distribution of monomer subunits in the polymer chain. The one of the most important copolymers of LA is poly(lactide-co-caprolactone), or poly(LA-CL). These homopolymers have contrasting physical and thermal properties: PCL exhibits good elasticity and permeability but poor mechanical characteristics (toughness), which is the opposite to PLA [21]. Based on research, it has been shown that, for the synthesis of effective initiators for the controlled ROP of lactide and cyclic esters, the following conditions must be met: (1) a metal center with high Lewis acidity to increase binding to LA; (2) M-X (M = metal, X = initiator, usually alkoxide or amide)—the alkoxide or amide moiety must be labile to react with alcohols; (3) the metal must be redox-inactive and inert to β-hydrogen abstraction, which otherwise can lead to chain termination with a loss of catalytic activity; and (4) in the LM complex, the binding of the metal to the ligand must be strong, which is necessary to fix single-site catalysis and prevent the formation of oligomeric metal particles. Based on these requirements, a large number of transition and basic metal complexes have been developed that exhibit high catalytic activity in both homopolymerization and copolymerization, as well as excellent stereoselectivity and the absence of side processes during polymerization, for example, transesterification reactions.

In this review, we paid special attention to metal complexes that have low toxicity and relative stability under polymerization conditions; complexes of rare earth metals will not be considered, since, despite their high efficiency and excellent stereoselectivity in ROP, the problem of their stability and the resistance of catalysts to large monomer loads still remains [22,23].

Depending on the nature of the initiator, there are essentially three types of mechanism that are considered for lactide ROP polymerization: (1) the anionic polymerization mechanism, (2) the coordination–insertion mechanism and (3) the activated monomer mechanism.

**Anionic ROP**

Suitable initiators for the anionic ring-opening polymerization of lactide are n-butyllithium and alkali metal oxides. Anionic polymerization is characterized by a fairly high rate of polymerization, and is also accompanied by a large number of side processes: chain termination and transesterification. At the beginning, the nucleophilic anion of the initiator attacks the carbonyl carbon atom of the lactide molecule; as a result, the CO single bond is cleaved. The resulting alkoxide in the next step is a new anion. When polymerization proceeds according to this mechanism, there is a risk of partial racemization if the initiator deprotonates the monomer or the chain end remains active.

**Coordination–insertion and monomer activated ROP**

Metal complexes that are active in ROP are characterized by two polymerization mechanisms: the coordination–insertion and activated monomer mechanisms. In accordance with the coordination–insertion mechanism, the active particle [M]-OR is first coordinated to the carbonyl oxygen atom of the cyclic monomer (lactide, glycolide, ε-caprolactone), and then it is inserted into the acyl–oxygen bond. In the case of non-alkoxide metal complexes, alcohol is added to form in situ initiating alkoxide species, leading to polymer chain propagation. At the last stage, the growing chain [M]-(O-C(O))OR is terminated, which leads to the formation of a polymer [24].

**Scheme 1:**
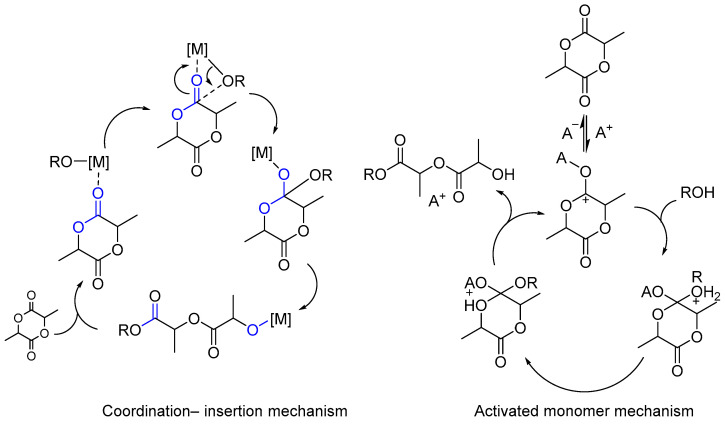
Coordination–insertion mechanism and activated monomer mechanism for the ROP of lactide.

In accordance with the “monomer-activated” mechanism, in the first stage, the metal atom is coordinated to the carbonyl oxygen atom, and then the ring opens under the action of an external nucleophile, which is usually an alcohol. The main difference between the mechanisms is that in the first case, the nucleophile, which opens the monomer heterocycle, is a ligand in the metal complex (which is why it is more correct to use the term “initiator” rather than “catalyst” for the metal complex); in the second case, the nucleophile is external (Scheme 1).

Controlled polymerization reactions such as live ROP make it possible to predict and control the physical properties of the resulting polymers. However, the live character of ROP can only be observed in very rare cases due to side reactions such as hydrolysis and transesterification chain transfer (also referred to as “backbiting” reactions). Intermolecular interesterification leads to a broadening of the molecular weight distribution, since intermolecular interesterification generates cyclic oligomers, which also leads to a decrease in the average molecular weight [24].

In some cases, controlled polymerization is achieved only with high loadings of initiators, which leads to the contamination of polymers with initiator residues and limits their practical use in production. An effective alternative to “living polymerization”, as well as a solution to the above-mentioned problems, is the so-called “immortal” ROP. The main difference between “immortal” ROP is the rapid and reversible growing polymer exchange between the active particles and the external nucleophile (chain transfer agent), thereby forming polymers with controlled molecular weights and a narrow molecular weight distribution. However, only a very limited number of catalytic systems that are capable of implementing “immortal” ROP are described in the literature, since for most ROP initiators, the chain transfer agents (alcohols) used in *i*ROP are poisons. Despite this, a number of effective catalytic systems that mediate “immortal” ROP have been described in the literature [24,25,26,27,28].

## 2. Group 1, 2 and 12 Metals (Li, Na, K, Ca, Mg, Zn)

An important task in the development of new catalytic systems is to increase the compatibility of the obtained polymers with the human body for the purpose of their further biomedical application. So, for example, the alkali metals sodium and potassium are non-toxic for the human body, and also participate in the metabolism of the human body. Lithium ions are involved in the metabolism of simple sugars, as well as the lipid and nucleic acid metabolism [29].

The polymerization of lactide under the action of n-BuLi, t-BuOK and t-BuOLi proceeds according to the anionic mechanism. At sufficiently high activity, polymerization is accompanied by a large number of side processes: chain termination and transesterification. The efficiency of the initiator is low due to the deprotonation reaction between *tert*-butoxide ion and L-lactide [30].

**Figure 2:**
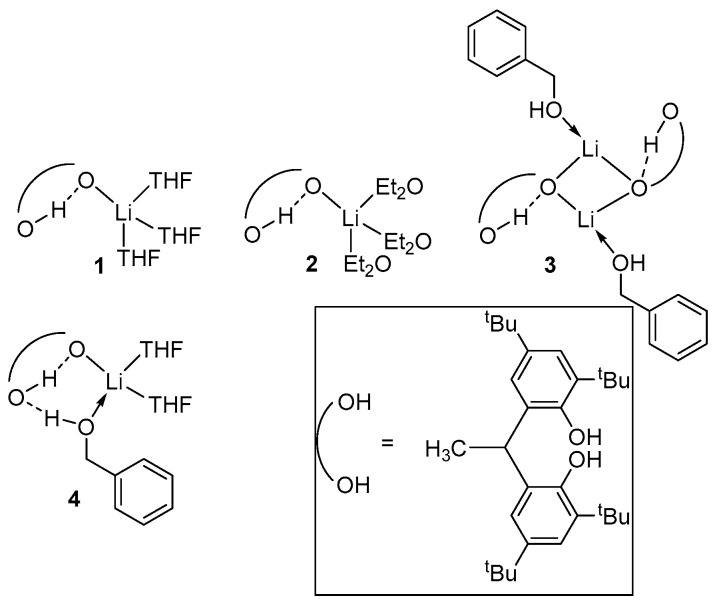
Lithium initiators **1**–**4** for ROP.

At the beginning of the 21st century, special attention was paid to binuclear lithium complexes and lithium complexes with phenolic type ligands, which showed high activity in lactide ROP in the presence of free alcohol (Figure 2) [31,32,33,34].

**Figure 3:**
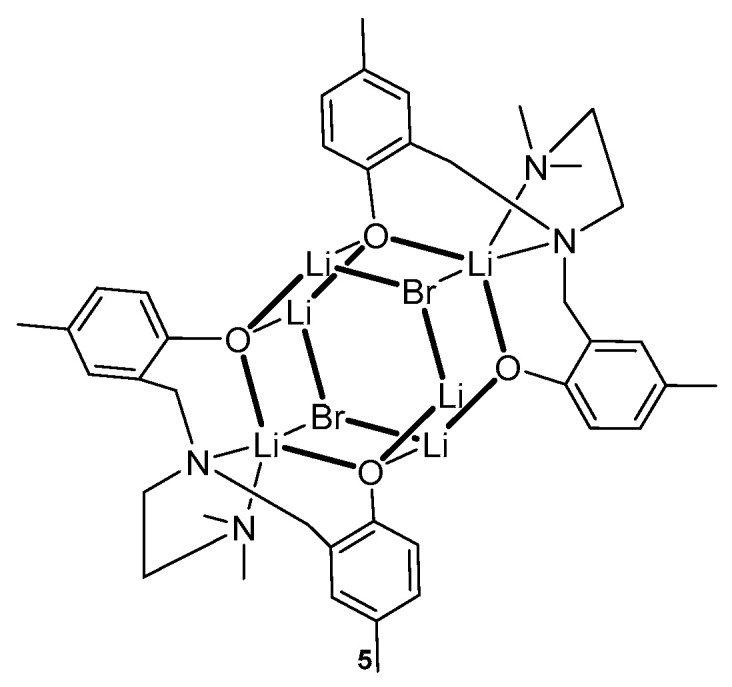
Lithium initiator **5** for ROP.

After the publication of reviews [34,35], several articles of a different focus were published. Kober et al. obtained high yields of lithium hexametallic complexes based on a diaminobisphenol ligand (Figure 3) [36]. Based on X-ray diffraction data, it was found that lithium complexes 1 and 4 in the solid state, regardless of the nature of the halide, have the same structural motif. Compound **5** in the presence of benzyl alcohol at room temperature (one equiv. BnOH per Li center) were found to be effective catalysts for L-lactide ROP to form moderate-molecular-weight PLLA. The low polydispersity index (*Đ*) of PLLA (*Đ*~1.05–1.07) indicates the controlled behavior of the polymerization of these complexes and a decrease in the occurrence of transesterification side reactions during the polymerization of cyclic ethers, which are characteristic of alkali metal compounds with less bulky ligands [37].

In addition, the coordination of BnOH with lithium centers has been proven to create four inactive species in the ROP of L-LA. Based on these results, as well as the analysis of the end groups, the authors suggested that polymerization is more likely to proceed via the activated monomer mechanism.

A number of non-toxic compounds of lithium, sodium and potassium iminophenoxide were synthesized in [38]. X-ray diffraction studies of their single crystals showed that these complexes are of a binuclear nature. All complexes exhibited moderate catalytic activity at 140 °C for the ROP of lactides without solvent in a reasonably controlled manner (*Đ*~1.05–1.16) to form PLA with a heterotactic bias (P_r_~0.72–0.83). An analysis of low-molecular-weight oligomers synthesized using some of the compounds showed that polymerization proceeds via the coordination–insertion mechanism, with the participation of the ligand as the initiating group.

For the resulting series of complexes, it was shown that complexes containing sodium exhibit a high catalytic activity with respect to ROP. Complexes containing potassium are less reactive than sodium complexes, while lithium complexes are less reactive. As a rule, an increase in Lewis acidity, as well as an increase in the size of the metal center atom, leads to an increase in reactivity. In this case, it turns out that for these complexes, the size of the central atom plays a key role in increasing activity.

On the contrary, for alkali metal complexes based on 2,2’-ethylidenebis(4,6-di-*tert*-butylphenol) as a ligand, a different order of activity in *rac*-LA ROP is observed: Li > Na > K > Rb. The relative order of stereoselectivity for this series of complexes in this system is Rb ≈ K > Na ≫ Li. The authors obtained the first example of the Rb complex in the ROP of *rac*-lactide, which showed moderate activity and good polymerization control (P_m_ = 0.71) [39].

In continuation of the study of alkali metal complexes based on amino-bis(phenolate) ligands, many of which demonstrated excellent catalytic activity in ROP, the authors of [40] reported the synthesis of new mono- and bimetallic complexes of sodium, lithium, potassium and calcium containing enriched electron tetradentate pyridylamino-bis(phenolato) ligands, as well as their activity in the ROP catalysis of *rac*-lactide in their pure form (as a melt) and in solution, both in the presence and in the absence of benzyl alcohol (BnOH). All complexes are active in the melt at 125 °C and in toluene at 25 °C. The lithium monometallic complex showed the best activity under melt conditions, while the sodium monometallic complex showed the highest conversion in solution at 25 °C. Based on the results of polymerization in solution, the order of reactivity of Na > Li > K > Ca can be established. The analysis of the end groups of the obtained polymers showed that, in general, the cyclic polymers were present in the low-mass region, regardless of whether the added alcohol was present or not. The activity of these complexes containing more-strongly electron-donating 2-*tert*-butyl-4-methoxyphenolate groups is much higher than the previously described alkali metal complexes containing 2,4-di-*tert*-butylphenolates [37].

**Magnesium complexes as initiators for ROP**.

**Figure 4:**
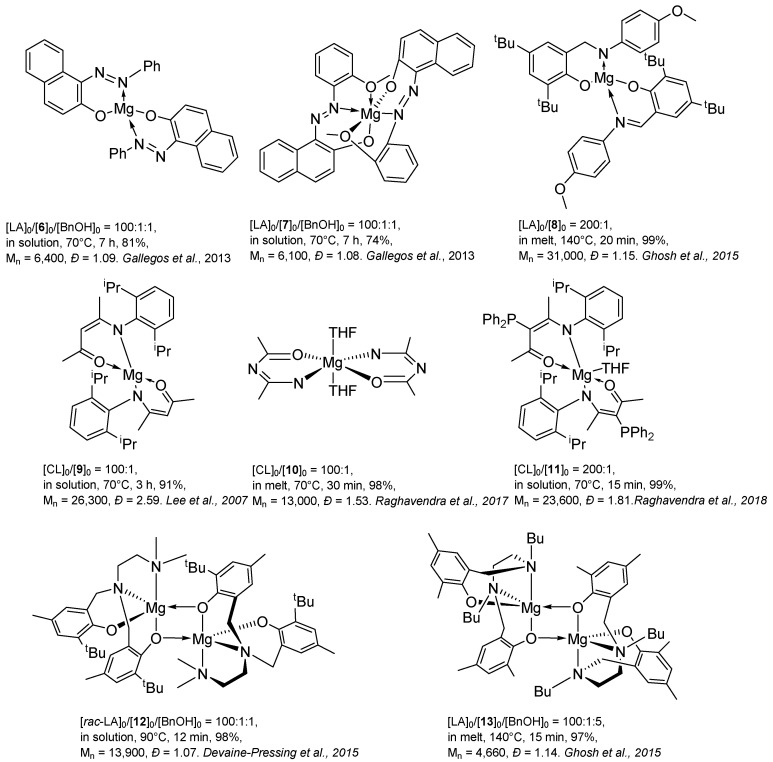
L_2_Mg complexes for ROP [47,48,49,50,51,52,53,54].

Magnesium complexes are good catalysts for use in the ROP of cyclic esters because the magnesium content in the ground is quite high. In addition, Mg complexes are strong Lewis acids. For ROP that proceeds via the coordination–introduction mechanism, the presence of a single-site catalyst structure is important [41,42,43,44,45,46] to provide a high degree of polymerization control. In general, L_2_Mg exhibits a significantly low catalytic activity with respect to the ROP of cyclic ethers. The currently known magnesium complexes with two ligands, such as Schiff bases (Figure 4, complexes **6**–**8**) [47,48,49], ketimines (Figure 4, complexes **9**–**11**) [50,51,52] and aminophenols (Figure 4, complexes **12**, **13**) [53,54], which have good catalytic activity in polymerization, are presented in Figure 4.

**Figure 5:**
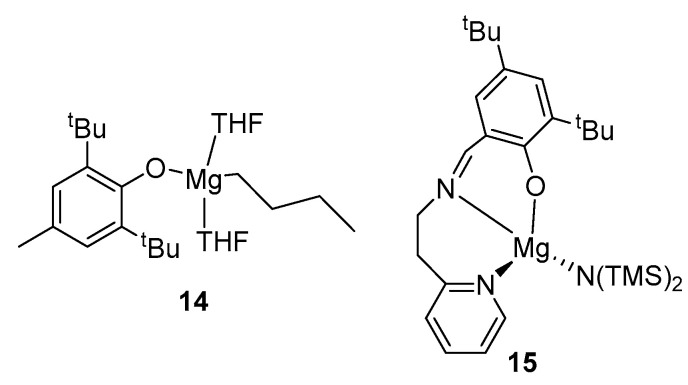
Magnesium complexes **14** and **15**.

One-site magnesium complexes show much higher activity in ROP. So, in the scientific group of Nifant’ev, the magnesium complex BuMg(BHT)(THF)_2_ **14** (Figure 5) was studied, which showed extremely high activity in the ROP of ε-CL ([ε-CL]:[BuMg(BHT)(THF)_2_] = 100:1, total monomer conversion in 1 min at 20 °C, M_n_ = 10,100, *Đ* = 1.39) [18]. Later, the polymerization of this complex in ROP LA was studied, in which it also turned out to be highly active ([L-LA]:[BuMg(BHT)(THF)_2_] = 100:1, 99% conversion, at 20 °C, M_n_ = 12,520, *Đ* = 1.97). However, this complex turned out to be unsuitable for the synthesis of L-LA and ε-CL random copolymers; during copolymerization, a slow homopolymerization of L-LA and almost no polymerization of ε-CL are observed [55].

An increase in the coordination number of magnesium, in the case of a magnesium complex based on phenoxyiminopyridine **15** (Figure 5), leads to a decrease in its activity, but at the same time, polymerization control increases ([L-LA]:[magnesium complex] = 100:1, conversion 33%, at 20 °C, M_n_ = 5300, PDI = 1.30) [56].

**Figure 6:**
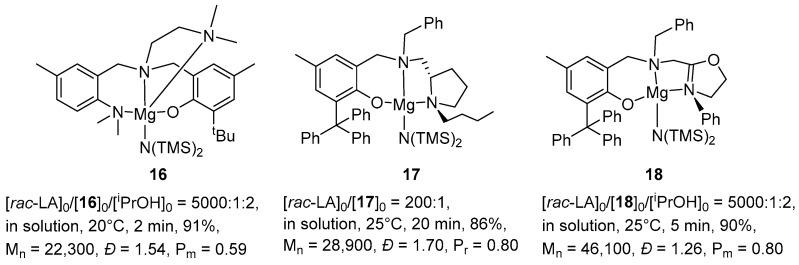
Aminophenolate magnesium complexes in the stereoselective ROP of *rac*-LA.

The vast majority of magnesium complexes, namely, those based on β-diketimines [57,58,59,60,61], trispirazolyl/trisindazolylborates [62,63], Schiff bases [64,65,66], heteroscorpionates [67,68] and aminophenolates [69,70,71,72,73,74], exhibit high heteroselectivity. Magnesium complexes that exhibit isoselectivity are less well described. Magnesium complexes based on tetradentate aminophenolate ligands (Figure 6, complex **16**) exhibit extremely high activity (TOF up to 135,750 h^–1^) and give PLA with a slightly isotactic bias (P_m_ = 0.54–0.60) and a high molecular weight (M_n_ up to 531 kg/mol) from *rac*-LA [72]. Magnesium complexes supported by tridentate chiral pyrrolidinylaminophenolate ligands (Figure 6, complex **17**) showed relatively high heteroselectivity (P_r_ = 0.80) and moderate activity against *rac*-LA ROP [69,70]. The introduction of chiral oxazolinyl aminophenolate ligands (Figure 6, complex **18**) led to an increase in the isoselectivity of the magnesium complex in the ROP of *rac*-LA (P_m_ = 0.80), an increase in the activity of the complex (TOF = 54,000 h^–1^) and leads to the formation of a polymer with a high mass (M_n_ = 461 kg/mol) [75].

**Zinc complexes as initiators for ROP**.

This section analyzes achievements since 2015, taking into account previously published reviews [43,76]. Zinc complexes combine two very attractive features: biocompatibility and high activity in ROP [77,78]. However, most of the existing zinc ROP initiators, except for the coordination complexes ZnX_2_→(neutral ligand)n (X = halogen, carboxylate) [79], have a serious drawback (instability compared to stannous octoate) due to the presence of unstable covalent bonds Zn–N or Zn–C in the molecule. At the same time, ZnX_2_→(neutral ligand)n complexes usually exhibit low activity as initiators. One of the ways to increase the stability of the catalyst is to expand the range of possible ligands that will be strongly bound to the zinc atom. Alcoholates containing nitrogen atoms to stabilize the monomeric or dimeric structure of the complex are able to bind with zinc through oxygen, forming moisture-resistant Zn–O bonds. Ligands of this type mainly include iminophenols, aminophenols, phenols containing a heterocycle, as well as ligands of a mixed type, where, for example, both an amino group and a heterocycle are present. It should be noted that heteroleptic zinc complexes (LZn–X, where X is a group suitable for migration from zinc atom to carbon atom in the first stage of the reaction) are usually more active than homoleptic zinc complexes (L_2_Zn). ZnEt_2_ and Zn[N(SiMe_3_)_2_]_2_ are the most commonly used reagents for the synthesis of zinc complexes with Et and the N(SiMe_3_)_2_ (HMDS) group at the zinc atom. The Et group is considered to be a poor initiator group due to its poor ability to migrate from zinc to carbon; thus, the addition of an external nucleophile (alcohol) is necessary to start the ROP process; zinc complexes containing the HMDS group are sometimes used without the addition of an external nucleophile due to its higher lability.

**Figure 7:**
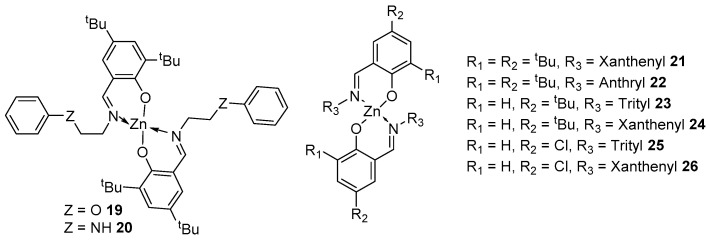
Zinc complexes **19**–**26**.

A fairly large class of compounds are zinc complexes based on iminophenols. It is worth noting that the observance of stoichiometry does not always help to achieve the desired result. In work [80], the reaction of ligands with ZnEt_2_ led to the formation of homoleptic complexes IIa-b, regardless of the reaction conditions—the temperature, solvent and molar ratios of ligand to metal of 2:1 or 1:1 (Figure 7). However, similar complexes of aluminum and calcium in this work turned out to be monoligands and showed high activity in ROP.

To understand the effect of substituents on the activity of the obtained homoleptic zinc complexes, the authors of the following work obtained six ligands with the reaction of ZnEt_2_, with which, in a ratio of 2:1, the following complexes were synthesized (**21**–**26**, Figure 7) with acceptable yields (37–52%).

Complexes **21**–**26** were tested as initiators of bulk *rac*-LA polymerization at 130 °C (Figure 7). At a monomer/initiator ratio of 100:1, complexes **21** and **23**–**26** in less than 60 min, as well as complex **22** in less than 3 h, provided high monomer conversion; the obtained molecular weights indicate an uncontrolled nature of polymerization. When using benzyl alcohol as a co-initiator under the same conditions, polymers with a molecular weight that is close to theoretical values are obtained. This means that the nucleophilicity of the phenolate ligand is lower than that of the alkoxide group, and the use of BnOH as a co-initiator makes the process more controllable. It is worth noting that in both cases, complex **22** has the lowest activity due to the too-large anthryl group, while **21** proved to be the best catalyst, providing moderate heteroselectivity (P_r_ = 0.60) and good polymerization control (*Đ* = 1.16) according to comparisons with other initiators [81].

**Scheme 2:**
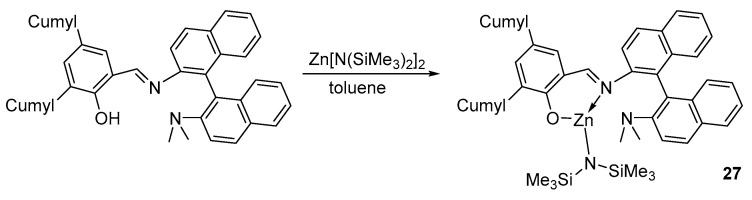
Synthesis of zinc complex **27**.

On the basis of the sterically loaded iminophenolate ligand, monoligand complex **27** was also obtained. The authors of [82] suggest that, due to the rigid binaphthyl framework, such ligands are more often bidentate, which, as a rule, leads to the formation of bis-ligand complexes, and only with bulky ortho-substituents is it possible to form the desired heteroleptic complex (Scheme 2).

The amide complex **27** showed high activity in the polymerization of *rac*-LA in toluene compared to similar magnesium complexes obtained in this work, but turned out to be less active in tetrahydrofuran. In the presence of isopropyl alcohol as a co-initiator and with an increase in temperature, the monomer conversion sharply increases from 24 to 96%. The dispersity of the obtained polycaprolactone varies from 1.19 to 1.70, and the P_r_ varies from 0.80 to 0.84, which indicates a high stereocontrol.

**Figure 8:**
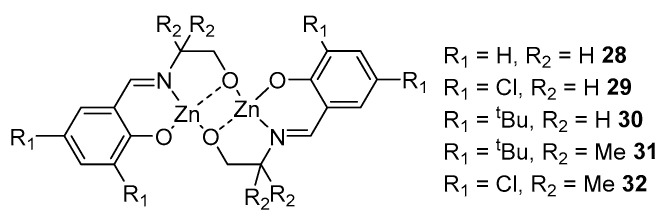
Zinc complexes **28**–**32**.

Salen-type ligands are also very popular and widely used in this direction. The authors of article [83] obtained five semi-salen-type ligands and binuclear zinc complexes based on them by reacting the ligand with an equimolar amount of ZnEt_2_, with subsequent heating to 80 °C for 10 h in a toluene solution (Figure 8).

In the framework of this reference, the catalytic activity of complexes **28**–**32** in the polymerization of lactide was also studied. Complex **28** showed the highest activity, with the lowest ratio of monomer to initiator, giving the highest conversion in less time compared to **29**–**32**. At a monomer/initiator ratio of 200:1, the conversion was 90% in 50 min, M_n_ = 13,900, *Đ* = 1.19 (polymerization conditions: in toluene solution, [M] = 0.5 M, 60 °C). Polymerization under the action of **28** can be described using a first-order equation, which, along with a narrow dispersity, confirms the controlled nature of the polymerization.

**Scheme 3:**
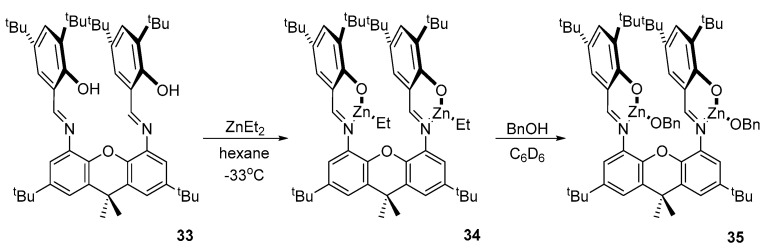
Synthesis of zinc complexes **34** and **35**.

It is generally accepted that the alkoxide group is more active in the ROP of cyclic esters than the alkyl group, so it is common practice to complex zinc with an alkyl group and then replace it with an alkoxide. The authors of article [84] obtained complex **34** through the standard exchange reaction between the ligand **33** and ZnEt_2_ at a reduced temperature with the elimination of ethane. Further treatments of compound **34** with benzyl alcohol in deuterobenzene led to the formation of the alkoxide complex **35** (Scheme 3).

The catalytic activity of **35** was tested in the ROP of *rac*-LA at 25 °C in a methylene chloride solution. Under these conditions, the polymerization of 600 equivalents of *rac*-LA reached a conversion of 86% after 24 h. Gel permeation chromatography (GPC) gave a rather narrow dispersity value of 1.23, indicating a controlled process, and homonuclear ^1^H NMR spectroscopy showed that **35** leads to a heterotactically inclined polylactide (Pr = 0.67), which is consistent with the data for related binuclear complexes. Polymerization experiments in toluene carried out at 70 °C showed higher activity but resulted in higher dispersity values and reduced heterotacticity.

**Figure 9:**
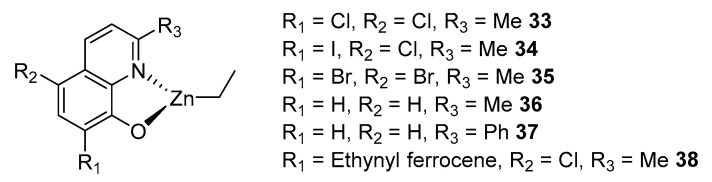
Zinc complexes **33**–**38**.

Heterocyclic compounds with one or more nitrogen atoms (pyridine, quinoline, triazoles, etc.) are also a good functional group for the design of ON-coordinating ligands. One of the simplest and most accessible compounds for these purposes is 8-hydroxyquinoline. In the following work [85], a number of modified hydroxyquinolines were obtained, which, when reacted with an equimolar amount of ZnEt_2_ in a toluene solution at room temperature, led to the formation of heteroleptic complexes **33**–**38** (Figure 9).

Compounds **33**–**38** proved to be quite effective polymerization initiators, the activity of which changes in the following order: **34** > **35** > **33** > **37** > **38** > **36**. All initiators show a linear increase in molecular weight with increasing conversion, and the dispersity is narrow in all cases (*Đ* < 1.10), which indicates the phenomenon of “living” polymerization. All complexes polymerize *rac*-lactide with a slight heterotactic slope (the maximum possible P_r_ = 0.70), and the degree of stereocontrol does not change with varying substituents in the R_1_ position, which excludes the possibility of “tuning” the stereoselectivity of the catalyst through this pathway. A slightly higher stereoselectivity was observed from CH_2_Cl_2_ to THF (P_r_ = 0.60 and P_r_ = 0.66, respectively, for **33**–**35**). An improvement in stereocontrol when using tetrahydrofuran as a solvent is also observed for other initiating systems, which is explained by an increase in the Lewis acidity of the catalyst due to the coordination of the solvent molecule with the metal [86,87].

**Figure 10:**
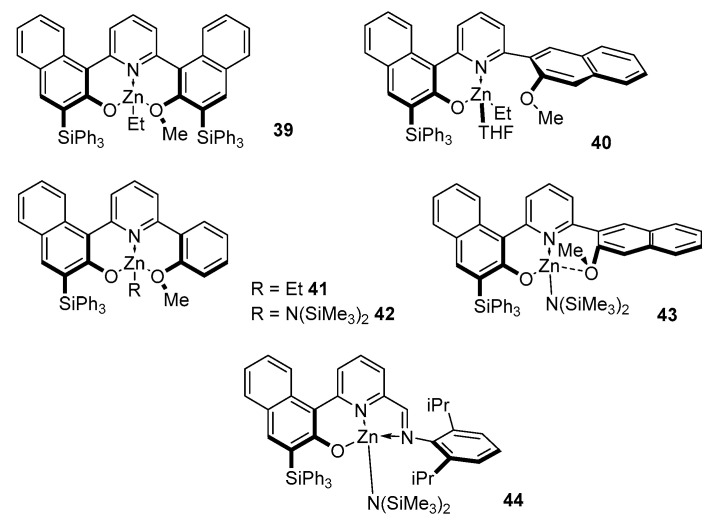
Zinc complexes **39**–**44**.

Pyridine is also widely used for the synthesis of such ligands, since its wide range of chemical properties makes it possible to obtain a rather complex molecule of the desired structure in just a few steps. The introduction of the binaphthol fragment increases the steric loading of the ligand and creates additional coordination with the metal atom, which are favorable conditions for the formation of monomeric heteroleptic complexes (Figure 10). Compounds **39**–**44** were obtained via a standard exchange reaction between the corresponding ligand and ZnEt_2_/Zn[N(SiMe_3_)_2_]_2_.

Complexes **40**–**41**, with less bulky substituents, showed higher activity than **39** and made it possible to convert 100 equivalents of *rac*-LA in a few minutes at 80 °C in toluene or 60 °C in THF, without the use of isopropyl alcohol as a co-initiator. The obtained weakly heterotactic polymers (P_r_ = 0.65–0.75) have a fairly wide dispersity (*Đ* = 1.5–2.1). As expected, the more nucleophilic amide complexes, **42**–**44**, are also active at room temperature, allowing for the conversion of 100–2000 equivalents of *rac*-lactide in a relatively short time. The polylactide obtained with **42** and **43** also has a fairly wide dispersity *Đ* = 1.3–2.0, which makes these initiators less effective and indicates a tendency toward transesterification side reactions. Complex **44,** even without isopropanol, gives polylactide with a narrow MWD (*Đ* = 1.11) and good agreement between the theoretical and experimental molecular weights. Moreover, the use of a co-initiator had a negative effect in cases **42**–**44**: at room temperature, these complexes were absolutely inactive.

To obtain heteroleptic nonpolymeric zinc complexes, the authors of [88] decided to increase the volume of pyridine alcoholate ligands by introducing adamantyl and cyclohexyl substituents into the carbon chain (using the previously described phenyl groups in the carbon chain [89] for comparison) and lengthening the carbon chain. The resulting zinc complexes were studied in the polymerization reaction of L-LA and ε-CL.

**Figure 11:**
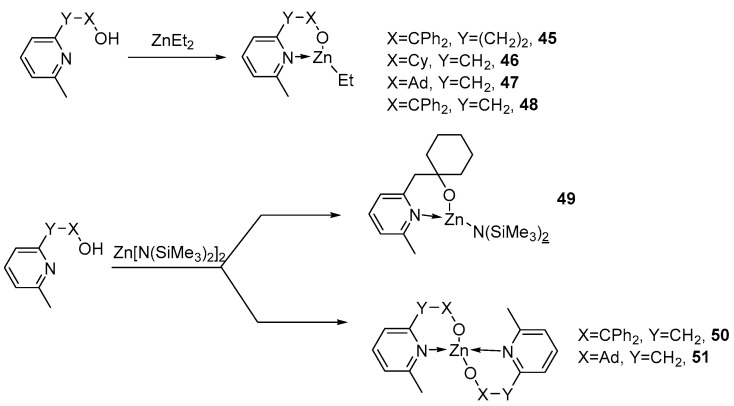
Zinc complexes **45**–**51**.

Three different types of zinc complexes (heteroleptic ethyl, amide and homoleptic) **45**–**48**, **49** and **50** and **51** (Figure 11) were studied in the polymerization of cyclic lactones: ε-Cl and l-La. All investigated substances were active in polymerization. Heteroleptic ethylzinc complexes **45**–**48** showed equally high activity in the presence of a benzyl alcohol co-initiator (BnOH), regardless of the type of ligand, converting 50 eq. of ε-CL to PCL in less than 15 min, but with poor reaction control for complexes **45**, **46** and **48** (PDI is 2.30–2.93). These results are indicative of intense transesterification occurring during polymerization. However, in the case of a sterically more hindered complex, **47**, ε-CL polymerization proceeds in a more controlled manner (*Đ* = 1.59).

The heteroleptic amide complex **49** showed a rather low activity in the polymerization of ε-Cl without the use of a co-initiator (BnOH). It was found that the polymerization of ε-CL in the absence of a co-initiator (benzyl alcohol) proceeds in a highly controlled manner (*Đ* below 1.2). The addition of benzyl alcohol significantly increased the catalytic activity of zinc complex **49**. The complete conversion of the monomer reached 30 min at room temperature; however, the resulting polymer has a fairly high *Đ* (2.12).

The heteroleptic zinc amide complex **49** and the ethylzinc complex **46** were also investigated in L-La polymerization. For the **49**/BnOH (1/50) system, complete conversion was observed within 2 h when the reaction was carried out in toluene at room temperature (*Đ* = 1.26). System **46**/BnOH exhibits moderate catalytic activity (80 °C, 39% conversion, 39 h, *Đ* = 1.19) [88].

**Scheme 4:**
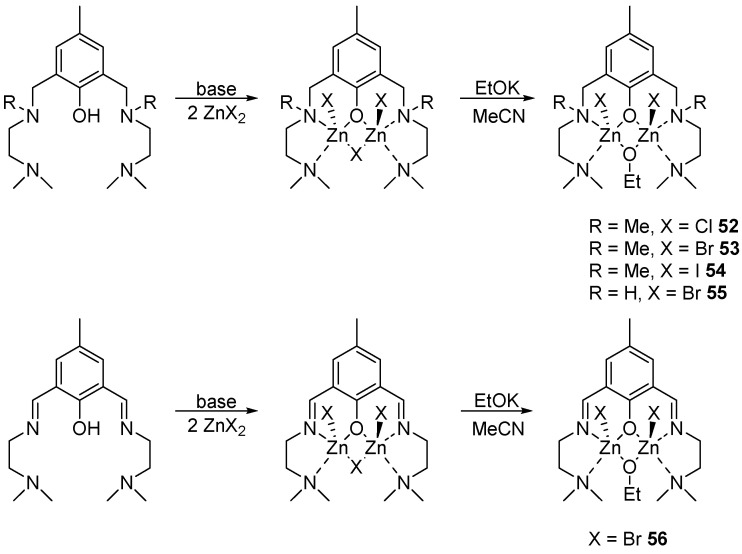
Synthesis of zinc complexes **52**–**56**.

Aminophenols are an extensive class of compounds that are successfully used as ligands for the synthesis of complexes of various metals and their further use in catalysis. The authors of [90] obtained ligands based on both aminophenols and iminophenols. Their further reaction with zinc halide in the presence of a base and subsequent treatment with potassium ethylate in acetonitrile gave binuclear complexes **52**–**56** (Scheme 4).

All complexes were tested in the polymerization of *rac*-LA in a solution of CH_2_Cl_2_ at room temperature and a loading of the monomer to the initiator at 200:1. Efficiency was compared in terms of the time required to achieve a 90% conversion. For the first three complexes, the activity turned out to be as follows: **53** > **52** > **54**, which, as can be seen, does not directly correlate with the electronegativity of the halogen. Since **53** showed the best result in this study, bromide complexes **55**–**56** were also tested in ROP: **56** showed very low activity, while **55** provided complete monomer conversion in 1 min. From this, we can conclude that the nature of the ligand has a rather strong effect on the rate: the slowest polymerization occurs as a result of using a rigid, electron-rich iminophenol, while the fastest polymerization is achieved in the case of a flexible amine ligand. Thus, **55** has the most potential for development, both in terms of speed and stereocontrol.

**Figure 12:**
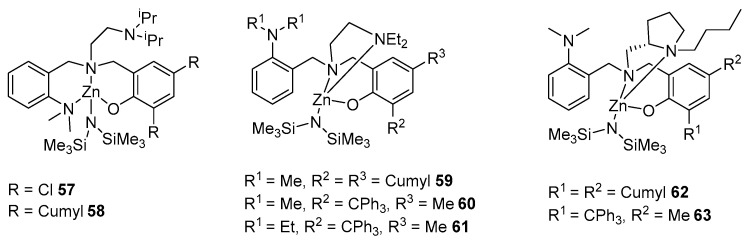
Zinc complexes **57**–**63**.

Another series of zinc amide complexes has been prepared and studied (Figure 12). Compound **59** in the solid state is a monomer, where the metal has an CN (coordination number) of four and is coordinated with the NEt_2_ group. The coordination environment of **58** is slightly different; in this case, the metal is coordinated by an arylamino group, while coordination with an alkylamino group is not observed, since zinc tends to have a tetracoordinate mode, if this is possible. Complexes **61** and **63** have a similar structure to **57**. The authors believe that, in the presence of the same alkyl substituents, the coordination ability of the alkylamino group is higher than that of the arylamino group, but an increase in the steric hindrance of the substituents in the alkylamino group can reverse the order.

All metal complexes proved to be effective initiators of *rac*-LA polymerization in toluene and THF solutions at room temperature and the ratio [M]:[I]:[^i^PrOH] = 200:1:1. The change in the catalytic activity in toluene can be represented as follows: **58** > **62** > **59** > **63** > **60** > **61** > **57**. It should be noted that the activity of all complexes decreases upon passing from toluene to tetrahydrofuran. The sterically loaded isopropyl group in **58** leads to the coordination of the metal with the arylamino group, and therefore, this initiator probably shows the best activity among the others (90% conversion in 20 min). However, **57**, with a similar metal coordination sphere but chlorine atoms in the ortho and para positions of the phenolate ring, takes 90 min to reach 88% conversion, all other things being equal, thus showing the lowest activity in the series [91].

**Figure 13:**
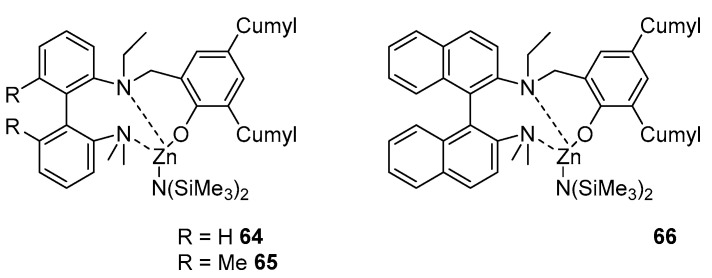
Zinc complexes **64**–**66**.

Zinc complexes **64**–**66**, obtained in the course of work [92], also have a CN of four, where the metal atom is chelated with three heteroatoms of the tridentate ligand and a bis(trimethylsilyl)amide group (Figure 13).

Compared with both similar magnesium complexes and complexes obtained in a previous work [82], these compounds show a significantly lower catalytic activity in the ROP of *rac*-LA, which changes as follows: **66** > **65** > **64**. Even in the presence of isopropanol, the resulting polylactide has a fairly wide MWD (*Đ* = 1.35–1.62); in most cases, the calculated molecular weight is much lower than expected. All these results indicate that the polymerization of *rac*-LA is poorly controlled by both magnesium and zinc complexes, which can be largely due to side processes of transesterification.

**Figure 14:**
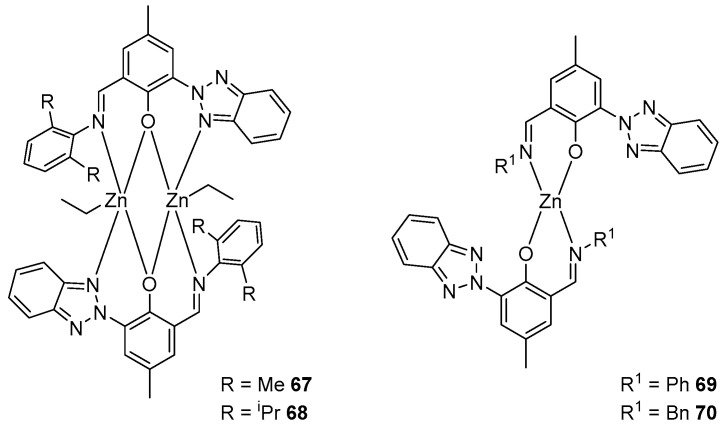
Zinc complexes **67**–**70**.

The treatment of the following mixed-type ligand with equimolar diethylzinc in a toluene solution at ambient temperature gives binuclear complexes **67** and **68**. However, the ligand, where R1 = Ph, gives a homoleptic mononuclear complex, **69**, in stoichiometric proportions, which can also be obtained with a ratio of reagents of 2:1 (Figure 14). Complex **70** was synthesized in a similar way to the latter. The activity of these compounds in the polymerization of lactide varies as follows: **67** > **68** > **69** > **70**. The difference in the activity of **69** and **70** indicates that effective resonance stabilization at R1 = Ph increases the Lewis acidity of the metal and, as a result, leads to a more efficient activation of the monomer at the metal center [93].

**Figure 15:**
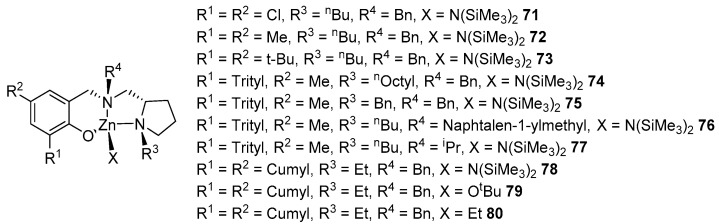
Zinc complexes **71**–**80**.

The authors of the following work investigated the influence of the steric effect of substituents on the structure and activity of the resulting metal complexes. Complexes **71**–**78** were obtained by reacting the corresponding ligand with zinc bis(trimethylsilyl)amide in a reactant ratio of 1:1 (yield 40–69%); the reaction of the ligand with one equivalent of ZnEt_2_ gave complex **80** (Figure 15). Complex **79** can be prepared by treating **78** with *tert*-butanol or via the metathesis of a related complex, where X = Cl, with potassium *tert*-butoxide, followed by the isolation of one equivalent of KCl. It should be noted that, during this reaction, a mixture of two diastereomers is formed, the ratio of which depends on the nature of the substituents; complexes **74** and **76** were obtained enantiomerically purely.

All complexes show themselves to be effective initiators of the polymerization of *rac*-LA in a solution of toluene or THF at room temperature, both as a single component and in the presence of isopropyl alcohol, leading to a polylactide with a moderate-to-high molecular weight and a narrow-to-broad MWD (*Đ* = 1.04–1.72). As in earlier studies [94], the activity of the complexes in toluene is higher than under the same conditions in tetrahydrofuran, especially for compounds with a bulky substituent at the R_1_ position. Complexes **71** and **72**, with small substituents in the ortho-position of the phenolic ring, show the highest activity. High conversions (94–99%) of the monomer can be achieved in less than 15 min at a ratio of [M]:[I]:[^i^PrOH] = 200:1:0. Replacing a chlorine or methyl group with a *tert*-butyl or cumyl group leads to a decrease in activity. This can be explained by the fact that bulky substituents located in the coordination sphere of the metal prevent the coordination/insertion of the monomer, which is an unfavorable factor for the use of such compounds in catalysis [95].

**Figure 16:**
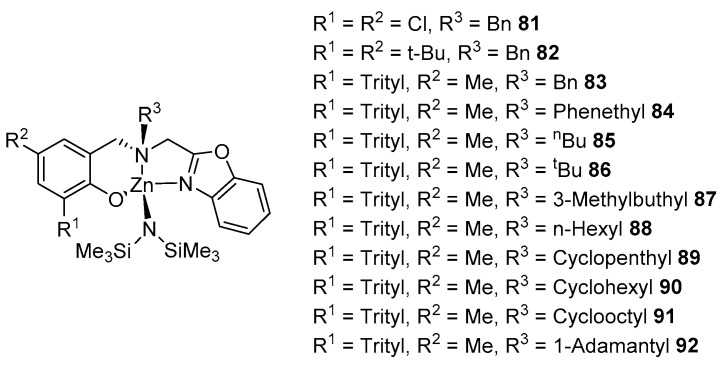
Zinc complexes **81**–**92**.

In continuation of the study, another series of related ligands and zinc complexes was obtained (Figure 16). These complexes are also good *rac*-LA ROP initiators and achieve deep monomer conversions in minutes or hours (depending on the functional groups in the ligand) at 25 °C. The resulting polymers have a high molecular weight, a narrow-to-acceptable MWD (*Đ* = 1.08–1.88) and are prone to isotacticity (P_m_ = 0.74–0.89). Complex **82** shows the best results among the entire series—at [*rac*-LA]:[I]:[^i^PrOH] = 500:1:1, a 90% conversion is achieved in 6 min, which, again, can be explained by the lower steric volume of the substituent in position R_1_, which does not block the access of the monomer to the metal center [96].

**Figure 17:**
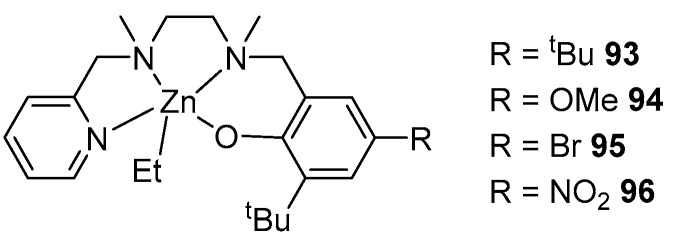
Zinc complexes **93**–**96**.

By reacting the tetradentate ligand with one equivalent of diethylzinc, complexes **93**–**96** were obtained (Figure 17). The treatment of these complexes with benzyl alcohol yielded similar alkoxide complexes in situ, which showed high activity in *rac*-LA ROP, with the rate changing as follows: **95**~**96** > **93** > **94**. In all cases, a >95% conversion is achieved in 6–10 min; however, even at these high speeds, process control is maintained, as evidenced by the GPC data (*Đ* = 1.06–1.17). There is also a tendency to form isotactic polylactide (P_m_ = 0.72–0.78) [97].

**Scheme 5:**
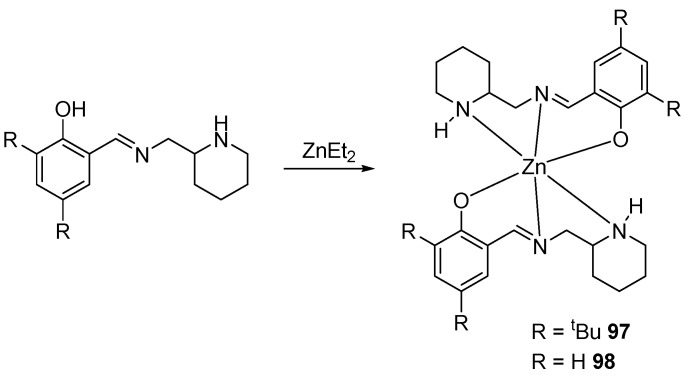
Synthesis of zinc complexes **97**–**98**.

Despite the stoichiometric ratio of ligand and metal, the authors [98] failed to obtain the desired heteroleptic complexes, and the homoleptic **97**–**98** were isolated instead (Scheme 5). Despite being sterically loaded, these compounds initiate *rac*-lactide polymerization well, converting 100 equivalents of the monomer with a 74 to 90% conversion in 5–15 min at 80 °C in a toluene solution. The resulting samples are characterized by a molecular weight that is close to the theoretical one, as well as a narrow MWD (*Đ* = 1.05–1.08).

**Figure 18:**
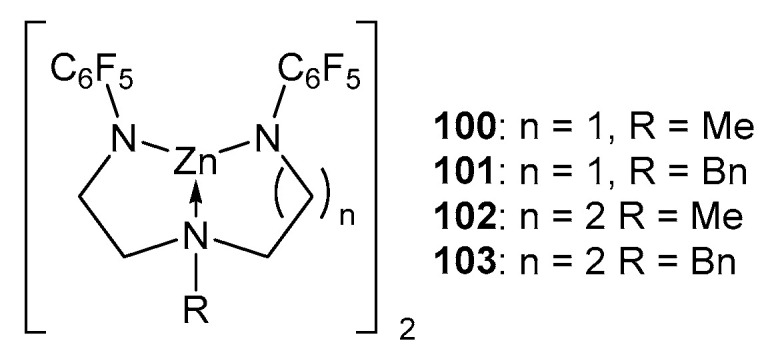
Zinc complexes **100**–**103**.

Only a few examples of zinc complexes with diamidoamine ligands are known in the literature (Figure 18). The complex [MeN(CH_2_CH_2_NSiMe_3_)_2_Zn]_2_ **99** was studied in the copolymerization of (D,L)-LA with glycolide and proved to be active, providing the complete conversion of monomers at 180 °C for 3 h. For example, from 702 and 398 equiv. lactide and glycolide, respectively, with respect to the catalyst, a polymer with M_n_ = 39,400 and *Đ* = 1.7 was obtained after 140 min [99]. In search of new active initiators, the authors of [100] chose zinc derivatives based on dialkylenetriamines, which contain C_6_F_5_, providing both steric protection and an increase in the positive charge on the zinc atom due to their acceptor properties. To determine the possibility of using the resulting complexes in ROP, compounds **101** and **103**, which have different lengths of carbon chains in the ligand structure, were tested in polymerizations of ε-CL and trimethylene carbonate (TMC). The authors chose bulk polymerization conditions, as they are the closest to those used in the industry and obtained encouraging results; namely, a >99% conversion was achieved at an organic monomer/complex ratio of 105:1 at 95 °C after 0.5 h. Polycaprolactone with high M_n_ and dispersity values was obtained using compounds **101** and **103**. A further increase in the monomer–initiator ratio led to high molecular weight polymers with a satisfactory *Đ* < 2. The polymerization results indicate that there is no significant difference in activity between complexes **101** and **103** and, therefore, no influence of an extra carbon atom in the ligand chain. The polymerization of TMC in the presence of complex **103** at a ratio of 1080:1 was completed after 60 min with the formation of a polymer with a molecular weight of 26,000 and a broad *Đ* = 2.46.

## 3. Group 4 Metals (Ti, Zr, Hf)

Most of the early results are described in reviews [76,101]. Titanium alkoxides are also effective and sought-after lactide ROP initiators due to their low toxicity, which minimizes problems associated with catalyst residues in commercial PLA products. However, initiators based on group 4 metals (Ti, Zr, Hf) were given little influence, probably due to the very oxophilic nature of M(IV), which leads to the formation of polynuclear complexes with alkoxy bridges in the absence of the stabilization of the central atom.

Advances in titanium complexes catalyzing the ROP of ε-CL show that PCL with desired properties can be obtained through the use of a number of auxiliary ligands, as with the ROP of lactide (LA) and ε-CL, as shown in Figure 1. Polymerization in ε-CL solution using titanium complexes with bidentate iminophenoxide ligands at 100 °C showed higher activity than tridentate iminophenoxide ligands [102]. However, the polymerizations were poorly controlled, with a wide dispersion. The bulk polymerization of ε-CL using titanium iminophenoxide complexes with [ε-CL]:[Ti] 200:1 at 80 °C resulted in PCL within 13 min [103,104]. However, the experimental molar masses were three times higher than the theoretical values, with relatively wide dispersion values. It was found that a number of titanium complexes supported on benzotriazole phenoxide ligands, first developed by the Ko group [105], are active for the polymerization of ε-CL in toluene at 80 °C, and the rate of polymerization of benzotriazole-phenoxide titanium complexes was lower than that of iminophenoxide titanium complexes. However, the actual molar mass of PCL was close to the calculated values based on two polymer chains growing on one metal center, and the polymerizations were alive.

As previously mentioned, the industrial PLA production process uses stannous octanoate as a catalyst at elevated temperatures (above the melting point of the monomer) and without a solvent. Tin octanoate is stable at these temperatures and produces high-molecular-weight PLLA. However, its use is limited by the toxicity of tin (the allowable concentration of tin in a polymer for biomedical applications is 20 ppm) [106] and the instability of the resulting PLA during melt processing is partly due to depolymerization under the action of the remaining active catalyst [107]. Thus, the search for catalysts that are a viable alternative to stannous octanoate, which should maintain their stability and production of high-molecular-weight PLLA at high temperatures, while being less toxic, is relevant. One such catalyst is represented by complexes of zirconium and hafnium based on aminotris(phenolate) ligands. These catalysts are extremely active towards L-LA at 180 °C, with catalyst loadings as low as 5 ppm for crude L-LA and 1 ppm for purified L-LA, and leads to high-molecular-weight PLLA (Lig_2_Zr(O-^i^Pr)(HO-^i^Pr):[cat/BnOH/LA] = 1/59/59.000, 80% conversion in 30 min, M_n_ = 55,700, *Đ* = 1.15, Lig_2_Hf(O-^i^Pr)(HO-^i^Pr):[cat/BnOH/LA] = 1/55/55.000, 71% conversion in 100 min, Mn = 52,700, *Đ* = 1.13.) They are based on zirconium or hafnium complexes of aminotris(phenolate) ligands. Notably, previous complexes of the same family have been extensively studied for lactide ROP; however, their activity was two to three orders of magnitude lower [108,109]. The authors explain this unexpectedly high activity with the nature of the substitution in the phenolate rings and, in particular, with the presence of ortho-aryl groups.

In [110], titanium complexes based on sterically bulky aminobisphenolate ligands were synthesized and structurally characterized. Complexes **104**–**105** were studied as initiators of ε-caprolactone ring-opening polymerization in the monomer melt at 100° C (**104**: [cat/ε-CL] = 1/300, 63% conversion in 60 min, M_n_ = 18,481, *Đ* = 1.36; **105**: [cat/ε-CL] = 1/300, 38% conversion in 60 min, M_n_ = 15,386, *Đ* = 1.32).

## 4. Group 13 Metals (Al, Ga, In)

**Aluminum complexes as initiators for ROP**.

Over the past 20 years, aluminum complexes stabilized with various ligands, such as salens or salans, iminophenols, ketiminates, pyrazolyl-containing compounds, bis-phenols with CH_2_, S, N, O-bridges and various derivatives of diethylenetriamines, due to their excellent stereoselectivity, oxophilic nature and Lewis acidity. In addition, unlike tin, whose toxicity is beyond doubt, the possible toxicity of aluminum derivatives has not yet been conclusively proven, although there are publications in the literature indicating a relationship between the incidence of neurodegenerative diseases in humans and an increased concentration of aluminum in the body. Most of the early results are described in reviews [11,111].

In particular, aluminum complexes based on NN and NO-type monoanionic bidentate ligands, such as phenoxyimine [112,113,114,115,116], amidinat [117] and pyrrolylaldimin [118] as initiators of the ROP of LA and CL, have been carefully studied; most of these complexes showed a high activity in ROP due to their less coordinatively saturated nature. It was found that the catalytic efficiency of these aluminum complexes is significantly affected by the steric and electronic properties of the ligand frameworks. Aluminum complexes based on NNN-, NON-, ONO-, OOO- and OSO-types of tridentate ligands have also been actively studied. Such ligands provide covalent bonding of the aluminum center with two nitrogen or oxygen atoms and an intramolecular interaction with one nitrogen (sulfur or oxygen) atom, which makes it possible, by varying the substituents (especially on the central nitrogen atom), to change the Lewis acidity of the aluminum atom.

**Aluminum complexes based on ON-type monoanionic bidentate ligands**

**Figure 19:**
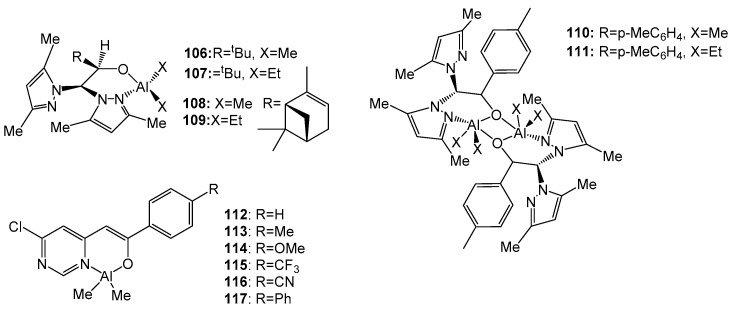
Aluminum complexes **106**–**117**.

The homoleptic complexes **106**–**111** (Figure 19) were synthesized via a reaction of the corresponding ligand and AlX_3_ (X = Me, Et) at a molar ratio of 1:1. In ROP, complexes **106**–**111** showed high activity (90–100% conversion at 100 °C and [M]:[I] = 200:1 ratio achieved within 10 min) [115].

The catalytic efficiency of the novel aluminum pyrimidylenolate complexes **112**–**117** (Figure 19) with respect to *rac*-LA ROP was evaluated in the presence of one equivalent of benzyl alcohol (BnOH). Polymerization was carried out at 70 °C in toluene ([LA]_0_:[Al]:[BnOH] = 100:1:1; [LA]_0_ = 0.83 M; [Al] = 8.33 mM; M_n_ (theoretically) = 14,400). Polymerization proceeded with high conversion within 9 h in all cases, except for the polymerization mediated by complex **116** (92% in 24 h). The dispersion values were relatively narrow (*Đ* = 1.11–1.20), indicating controlled living polymerization. An analysis of the microstructure of the polylactides, determined by examining the methine region of the homonuclear decoupled ^1^H NMR spectra of the obtained PLAs, showed that, in all cases, PLAs were formed with isotactic bias and P_m_ values in the range of 0.65–0.70. Complexes **112**–**117** were also studied in ε-CL ROP. Polymerization was carried out in toluene at 70 °C; the molar ratio [ε-CL]_0_:[Al]:[BnOH] was fixed at 100:1:1 ([Al] = 12.50 mM; [ε-CL]_0_ = 1.25 M). Narrow dispersity values were also observed in all cases (*Đ* = 1.10–1.23), indicating the controlled and living polymerization of ε-CL. It is clearly seen that the polymerization of ε-CL, mediated by all aluminum complexes, exhibits a significantly higher activity compared to *rac*-LA ROP. The polymerization reached a high conversion (>93%) in 10 min. It was found that complex **115** is the most active initiator of ε-CL polymerization, since a 95% conversion is achieved in just 5 min [119].

**Figure 20:**
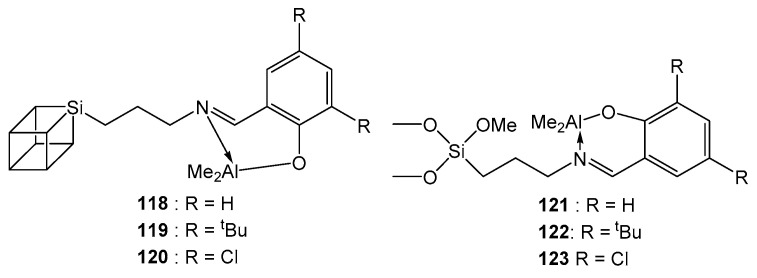
Aluminum complexes **118**–**123**.

Complexes **108**–**123** (Figure 20) showed similar activity in ROP, having been tested as *rac*-LA polymerization initiators. All studied compounds showed good catalytic activity; the conversion was 99% at 80 °C in 24 h, at a ratio of [M]:[I] = 100:1. The good agreement between M_theor_ and M_GPC_ and the production of a polymer with a narrow *Đ* indicate the controlled nature of the polymerization. The GPC data show only one polymer chain growing from the metal center [120].

**Figure 21:**
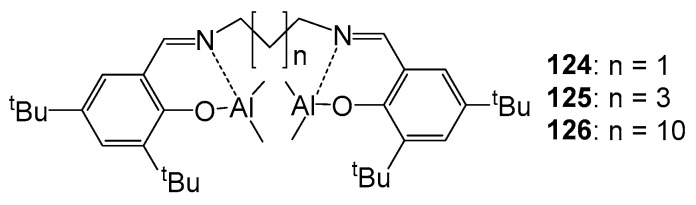
Aluminum complexes **124**–**126**.

The activity of homoleptic complexes **124**–**126** (Figure 21) in the ROP of *rac*-LA was studied. The polymerization was carried out in a toluene solution at 70 °C in the presence of four equivalents of isopropanol as initiators, so the exchange of alkyl groups for more nucleophilic alkoxy groups takes place in situ. The use of **124**–**126** leads to obtaining a monomodal polymer with a narrow MWD (1.09 ÷ 1.20), as well as a M_GPC_ close to M_theor_, which indicates polymerization of a controlled nature. Compound **124** turned out to be the most effective; monomer conversion was 100% within 12 h and *Đ* = 1.09, which is explained by the proximity of metal centers in the **124** bimetallic system. The stereoregularity of PLA prepared with **125** and **126** was significantly lower (P_m_ = 0.56 and 0.59, respectively) than that of PLA prepared with **124** (P_m_ = 0.82). This is consistent with the reduced steric environment around the reaction centers, and is also due to the greater flexibility of the coordination sphere imparted by the longer alkyl bridges [121].

**Aluminum complexes based on ONO-type ligands**

**Figure 22:**
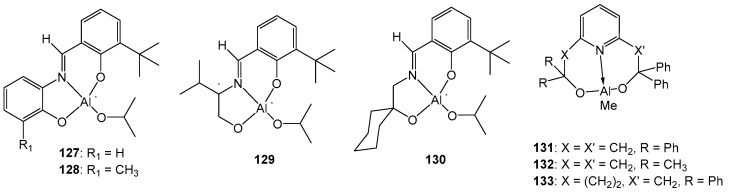
Aluminum complexes **127**–**133**.

In order to compare the properties of complexes with and without chirality, semi-salen ligands and complexes **127**–**130** (Figure 22) were prepared. The study of the activity of **127**–**130** in D,L-LA ROP was carried out in a toluene solution at 70 °C and the ratio [M]:[I] = 100:1. Under these conditions, the obtained compounds showed moderate activity—a conversion of 21–80% was achieved in 96 h, which is worse than the activity of the previously studied salen complexes based on aluminum. The use of pyridine as a solvent greatly increases the activity, reducing the polymerization time to 3–24 h, and also increasing the conversion values. Further study of the structures of the obtained polymers revealed the formation of isotactic blocks of the copolymer when using complexes with a chiral metal center (P_m_ = 0.92) [122].

**Figure 23:**
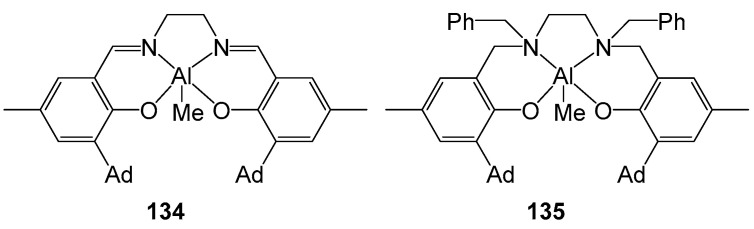
Aluminum complexes **134**–**135**.

In [123], the authors reported the synthesis of a series of new aluminum complexes based on 2,6-bis(hydroxyalkyl)pyridines and their use in the ring-opening polymerization of L-LA and ε-CL. Compounds **131**–**133** (Figure 23) were synthesized using the reaction of AlMe3 and ligands in a 1:1 ratio at −30 °C with good yields (82–97%). Using DOSY NMR, the dimer–monomer equilibrium of complex **133** was established in DMSO and chloroform solutions. The use of **131**–**133** in the polymerization of L-lactide showed a high catalytic activity of the obtained compounds. A conversion of >95% is achieved in 4–27 h at a ratio of [M]:[I] = 50:1. The activity decreases in the order **131** > **132** > **133**, which is explained by the increase in the steric size of the substituents from **131** to **133** [123].

**Aluminum complexes based on ONNO-type ligands**

The authors of one study [124] obtained complexes **134**–**135** (Figure 23) by mixing free ligands with an equimolar amount of AlMe_3_ in a toluene solution at 110 °C for 24 h. The measurement of the catalytic activity of the obtained compounds was carried out in a solution of toluene at 70 °C. The use of **134**–**135** in the polymerization of *rac*-LA leads to the production of an isotactic polymer (P_m_ = 0.88) with a narrow MWD and a conversion of more than 90% within 24 h, which indicates a good catalytic activity. However, the presence of sterically loaded substituents leads to a decrease in catalytic activity compared to similar systems.

**Figure 24:**
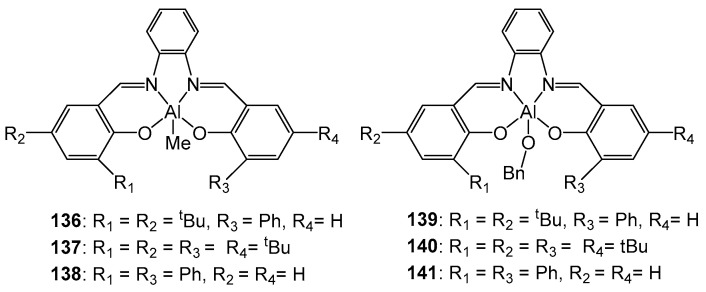
Aluminum complexes **136**–**141**.

The authors of article [125] obtained complexes **136**–**138** (Figure 24) by reacting the corresponding ligands with a stoichiometric amount of AlMe_3_ at room temperature. The study of the activity of **136** in the polymerization of *rac*-LA revealed a low activity of the compound. To increase the activity, the resulting compounds were reacted with BnOH to give complexes **139**–**141**. The modified complexes showed a significantly higher activity in the polymerization of *rac*-LA; in addition, their use led to controlled polymerization.

**Diamidoamines** are another type of ligand that allow for the precise control of the catalytic properties of a complex by varying the Lewis acidity and the steric availability of the metal atom. This can be achieved by using various substituents on the nitrogen atoms. Aluminum complexes supported with diamidoamine ligands have been reported to have catalytic activity in *rac*-LA ROP. Typically, changes in the number of carbon atoms in the ligand can lead to changes in the metal coordination polyhedron, and thus to changes in the catalytic activity [126,127].

**Figure 25:**
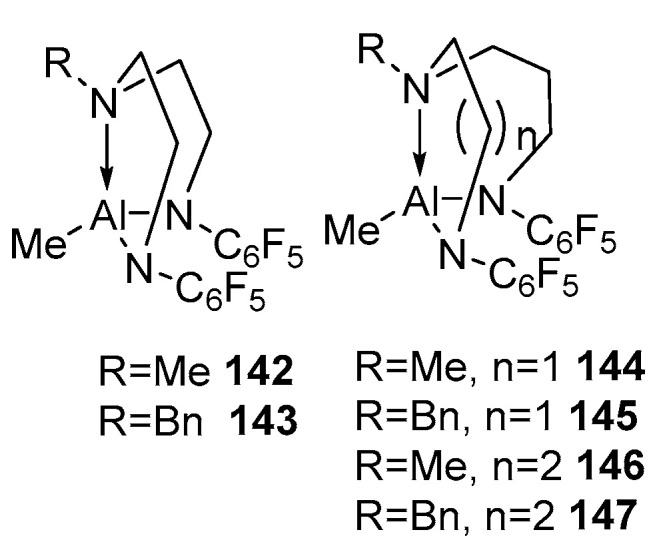
Aluminum complexes **142**–**147**.

In [128], the authors obtained a number of aluminum complexes based on ligands of the diamidoamine type with different numbers of carbon atoms in the ligand (Figure 25). Preliminary tests were carried out to study the catalytic activity of complexes **143**, **144** and **146** with regard to such cyclic lactones as L-LA and ε-CL. The activity of complexes **143**, **144** and **146,** which contain different numbers of carbon atoms in the ligand structure (2.2; 2.3; 3.3), was practically the same. A somewhat higher activity of **143** ([M]:[I]:[BnOH] = 50:1:1.05, in toluene solution, 80 ° C, 99% conversion in 1 h, M_n_ = 4,400, *Đ* = 1.15) can be noted as compared to **144** and **146**. In this case, the conversion values change significantly within 1 h, and the reaction rate increases in the series **143** (2.2) > **144** (2.3) > **146** (3.3). The authors attribute this to the formation of a pre-reaction complex, from which the reaction begins, and the rate of formation of which depends on the structure of the initiator.

**Indium complexes as initiators for ROP**.

Recently, some indium compounds have been shown to promote the ROP of lactide [129,130,131,132,133,134,135,136,137,138,139], ε-caprolactone [140] and β-butyrolactone [141], but relatively little research has been carried out in this area. Thus, simple mixtures of indium trichloride, benzyl alcohol and triethylamine have been shown to catalyze the ROP of *rac*-LA to form a highly heterotactic polylactide [137]. Indium-based catalysts are attractive due to their novel reactivity profile, low toxicity and stability in water.

**Scheme 6:**
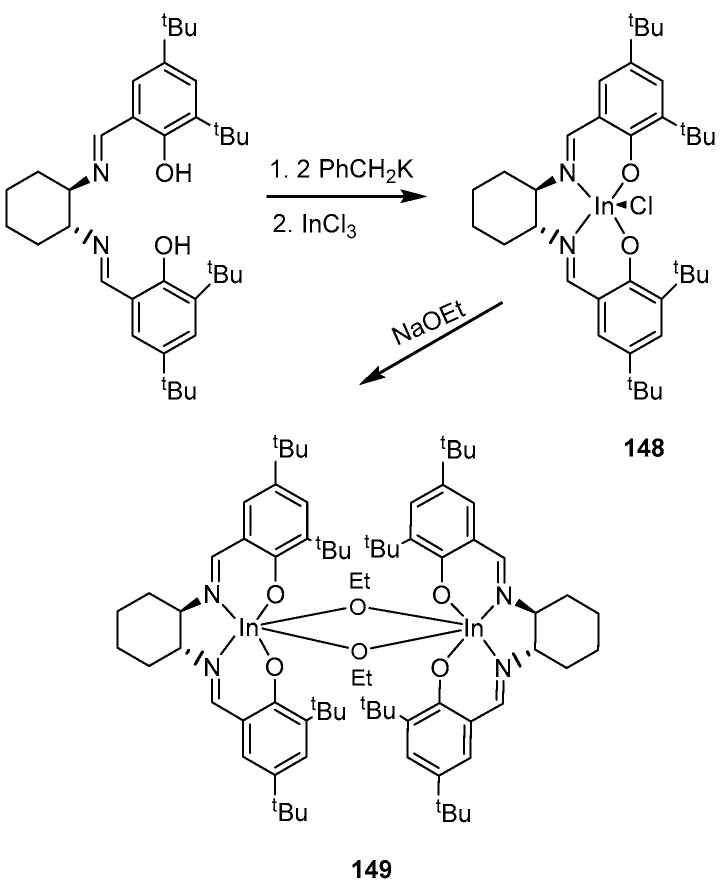
Synthesis of indium complexes **148**–**149**.

The preparation of indium chloride complexes based on a salen-type ligand is described in [133]. At the first stage of the synthesis of the target complexes, the authors deprotonated racemic or enantiomerically pure N,N’-bis(3,5-di-*tert*-butylsalicylidene)-1,2-cyclohexanediamine with two equivalents of PhCH_2_K, then added 1 equiv. InCl_3_, which led to the corresponding racemic or enantiomerically pure indium chloride derivatives [ONNO]InCl (*rac*- or (R,R)-**148**) (Scheme 6). The further interaction of [ONNO]InCl (*rac*- or (R,R)-**148**) with one equivalent of NaOEt leads to the formation of [ONNO]In(OEt) alkoxide complexes (*rac*- or (R,R)-**149**).

The authors of [133] tested the *rac*- or (R,R)-**149** alkoxide complex as an initiator in the *rac*-LA ring-opening polymerization reaction (30 min, CH_2_Cl_2_, 25 °C, 97% conversion). The resulting polymer is isotactic (P_m_ = 0.77). The polymerization of 1000 equivalents of *rac*-LA with *rac*-**149** is completed in less than 4 h and shows high selectivity.

**Figure 26:**
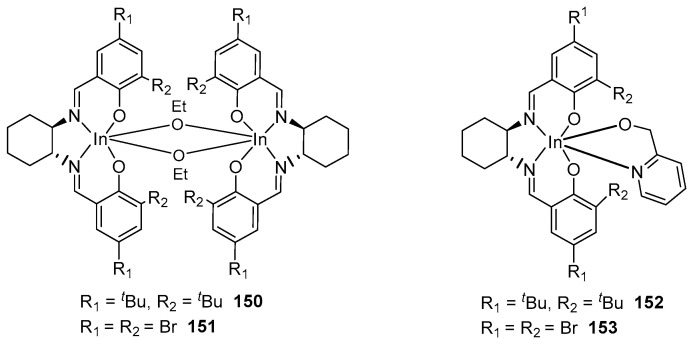
Indium complexes **150**–**153**.

By replacing OEt in alkoxide complexes **150** and **151** with 2-methoxypyridine, the authors of [142] managed to obtain monomeric complexes **152** and **153** (Figure 26) due to the additional coordination of the pyridine ring to the indium atom. The polymerization activity of the monomeric and dimeric complexes **150** and **152** is identical; both have similar activity and isoselectivity (P_m_∼0.75). In contrast, the dimeric analog of the less-bulky **151** catalysts have a longer initiation period, which is completely minimized for their monomeric analogs. These observations confirm that the initiation of LA polymerization first requires the dissociation of the dimers.

**Scheme 7:**
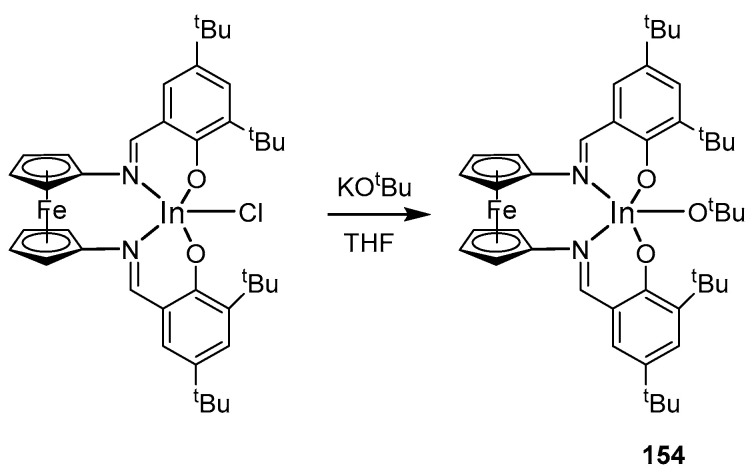
Synthesis of indium complex **154**.

The synthesis of another example of an indium alkoxide complex, (salphene)In(OtBu) **154**, was described in [143]. The target alkoxide complex was obtained via the equimolar interaction of (salphene)InCl and KO^t^Bu (Scheme 7). The indium chloride complex was obtained earlier from InCl_3_ and K_2_(salphene). It should be noted that the authors used KH as a base for the synthesis of the potassium salt of the K_2_(salphen) ligand.

The authors evaluated the activity of compound **154** in the ROP of various cyclic esters (L-LA; D,L-LA; TMC; ε-CL; δ-VL; and β-BL). The alkoxide complex (salphene)In(OtBu) showed high activity under mild conditions in the polymerization of lactones, giving extremely-high-molecular-weight polymers ([I] = 0.005 M, 2 min, toluene, 25 °C, *Đ* = 1.28, 99% conversion).

**Indium complexes based on NNO- and NO-type ligands**

**Figure 27:**
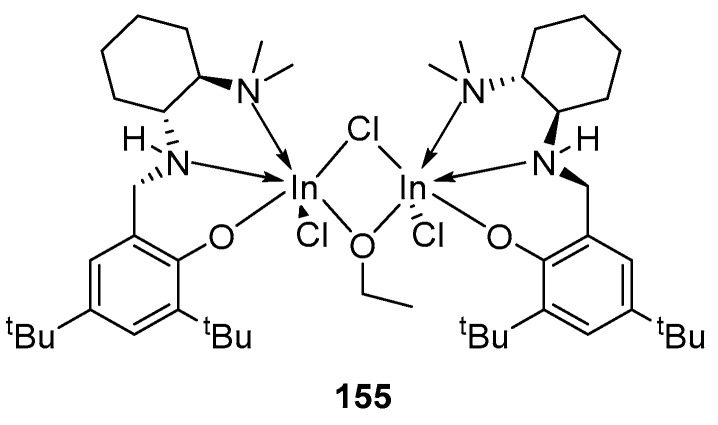
Indium complex **155**.

Until 2010, only two examples of well-defined indium complexes were known; **155** catalyzes *rac*-LA ROP, showing moderate isoselectivity (Figure 27) [130,134].

**Scheme 8:**
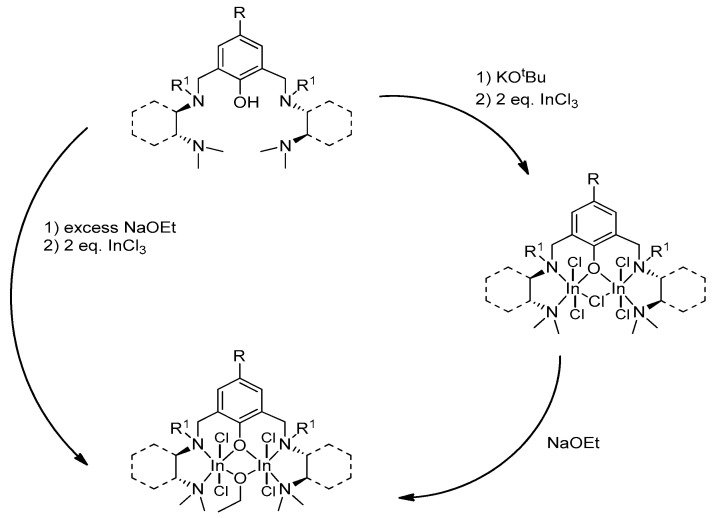
Route for the synthesis of indium alkoxide complexes based on ligands L_1_ and L_2_.

**Scheme 9:**
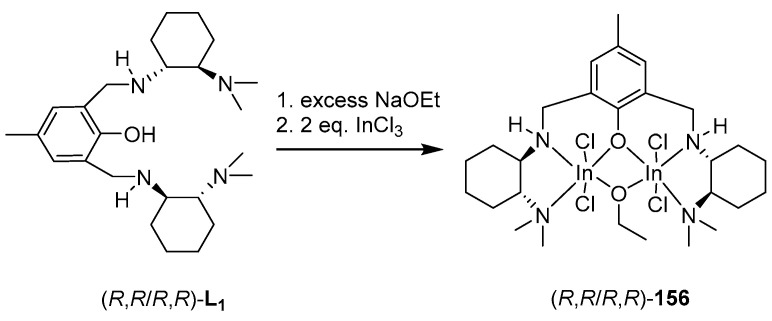
One-pot synthesis (*R*,*R*/*R*,*R*)-**156**.

**Scheme 10:**
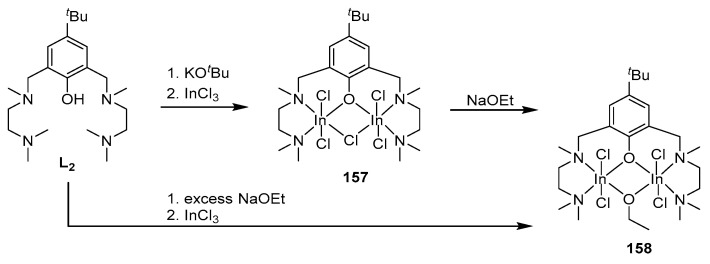
Synthesis of indium complexes **157** and **158**.

In continuation of these studies, the authors of [144] performed the synthesis of indium alkoxide complexes based on ligands L_1_ and L_2_. Target complexes were obtained in two ways (Scheme 8 and Scheme 9). The reaction to obtain alkoxide complexes **156** and **158** was carried out either with the isolation of the intermediate halide, which then reacted with NaOEt, or without isolation (one-pot synthesis); in this case, NaOEt itself acted as the base (Scheme 10) [130,145,146].

The authors studied the activity of the resulting complexes (R,R/R,R)-**156** and **158** with respect to lactide. The catalytic activity of complex **158** (97% conversion in 8 days) was higher than that of **156** (82% conversion in 11 days) (CH_2_Cl_2_, 25 °C, P_m_ = 0.87 and 0.89, respectively), but both complexes show higher selectivity and lower activity as catalysts for *rac*-LA ring-opening polymerization compared to similar monomeric indium complexes [130,132,133,146].

**Scheme 11:**
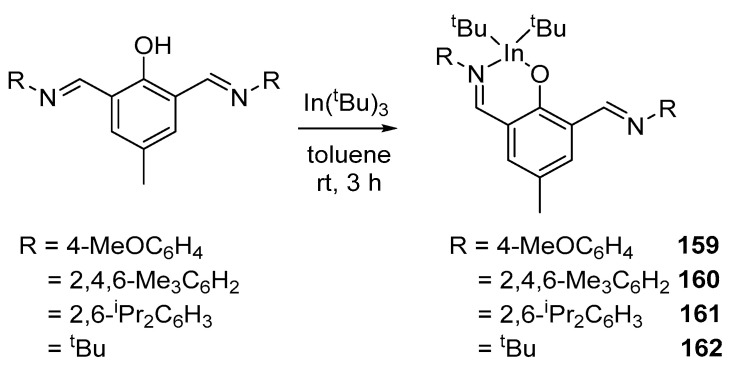
Synthesis of indium complexes **159**–**162**.

The interaction of tri-*tert*-butylindium with substituted bis(imino)phenoxide ligands in a ratio of 1:1 led to the formation of heteroleptic complexes **159**–**162** (Scheme 11) [147].

Ghosh et al. also studied the catalytic activity of indium bis(imino)phenoxide complexes with respect to various lactide stereoisomers. Polymerization was carried out in a monomer melt (*rac*-LA or L-LA) at 140 °C. According to the results, the catalytic activity decreased in the following order: **162** > **161** > **160** > **159**, which generally corresponds to a decrease in steric hindrance due to substituents on the nitrogen atom; the monomer conversion reaches 90–95%. The resulting polymers are characterized by a very narrow molecular weight distribution (MWD), and all PLAs obtained are isotactic in nature (P_m_ = 0.70–0.76). However, the activity of indium complexes is still lower than that of similar gallium complexes.

Later, the authors of [148] studied the role of phenolate substituents and the chirality of the tridentate ligand system of binuclear indium alkoxide complexes in the ring-opening polymerization of *rac*-LA. In contrast to the previously described dihalide complexes, the dichloride complexes discussed in this article, namely the (±)-(NNOSiPh_3_)InCl_2_ (**163**) and (±)-(NNOAd)InCl_2_ (**164**) complexes, were dimeric in the solid state, forming heterochiral (R,R/S,S) dimers through the chloride bridge of the ligand.

**Scheme 12:**
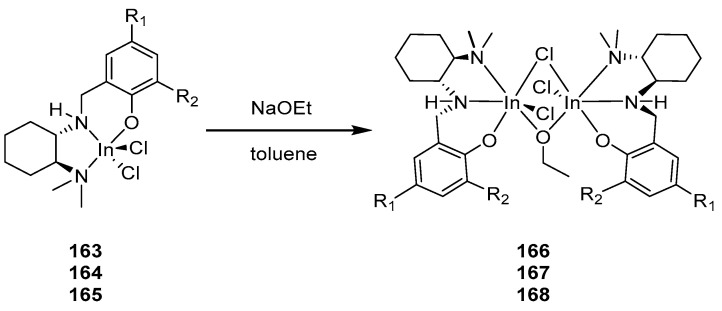
Synthesis of indium complexes **163**–**168**.

Binuclear complexes (R,R)—[(NNOR)InCl]_2_(μ-Cl)(μ-OEt) (R = SiPh_3_ (**166**); Ad (**167**); Cm (**168**)) were obtained through the direct treatment of indium chloride complexes with sodium ethylate (Scheme 12). The (R,R)-**168** complex was not isolated in its pure form due to the insufficient purity of complex **165**.

The polymerization of *rac*-LA using catalysts **166**–**168** showed that, in all three cases, polymers with controlled molecular weights and low polydispersity values are obtained. The adamantyl- and cumyl-substituted compounds **167** and **168** had similar stereoselectivity, giving isotactically enriched PLA (P_m_~0.6). On the contrary, the silyl-substituted analogue of **166** was less stereoselective, with a racemic catalyst, forming atactic PLA (P_m_~0.5).

**Figure 28:**
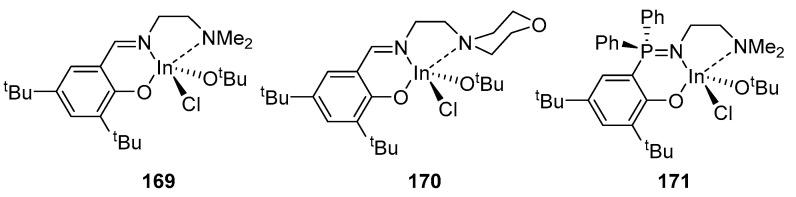
Indium complexes **169**–**171**.

The monoalkoxide complexes **169**–**171** were tested in *rac*-LA ROP (Figure 28). The polymerization of *rac*-LA under the influence of complexes **169**–**171** proceeded in a controlled manner (*Đ* = 1.02–1.10); complete conversion of the monomer was achieved in several hours at room temperature. Complexes **169** and **171** showed higher activity than complex **170**; the authors explain this based on the presence of a morpholine side group in complex **171**, which actively competes with the monomer for the coordination site. Complexes **169**–**171** show no signs of transesterification during polymerization. The resulting polylactides turned out to be atactic; only in the case of complex **170**, polymerization proceeded with a slight atactic shift (P_r_ = 0.63). Complexes **169**–**171** showed higher activity in the polymerization of ε-CL (complete monomer conversion was achieved in 20–40 min at room temperature) than for *rac*-LA. However, along with the high activity, the ROP control was significantly reduced [149].

**Gallium complexes as initiators for ROP**.

The chemistry of gallium attracts more and more interest from both scientists involved in fundamental research and from industry, due to the general lack of knowledge of gallium derivatives, which, however, demonstrate a number of useful properties that find practical applications. One of the main areas of application for gallium compounds on an industrial scale is the use of gallium arsenide as a semiconductor, which functions as a part of expensive high-efficiency solar cells, detectors and light-emitting devices [150]. Research is ongoing in this area, aimed at finding new, more efficient precursors containing gallium, as well as at optimizing methods for obtaining these compounds. The synthesis of new gallium derivatives, which are promising as biologically active substances in pharmaceuticals, especially in radiopharmaceuticals, is another important direction in the development of the chemistry of gallium compounds. The antitumor properties of gallium compounds were first described in 1971 by Hart et al. [151]. The activity of gallium derivatives as medicinal compounds is based on the similarity of the chemical properties of Ga(III) and Fe(III) salts, which explains the ability of gallium to form complexes with proteins and ligands that bind the iron transport protein in the blood [152]. On the other hand, ^67^Ga in the form of radiopharmaceuticals with a long half-life is used as a widely used diagnostic tool in the diagnosis of certain types of cancer, as well as infectious and inflammatory diseases, in positron emission tomography (PET) [153]. It is important to note that Ga(III) salts have a low toxicity profile because they are not metabolized by living organisms when ingested or inhaled [153].

The low toxicity of gallium derivatives opens up new horizons for the use of these substances. One such application is the synthesis of biodegradable polymers using metal complexes as ROP initiators of cyclic esters. Today, the design of the structure and the preparation of less toxic initiators based on non-transition-metal complexes (compared to those currently used on the basis of tin), which at the same time exhibit high catalytic activity in the synthesis of these polymers, is one of the important tasks of modern chemistry. Gallium derivatives are very attractive from this point of view, since they are Lewis acids, and due to the oxidation state of +3 and the coordination number reaching six, they have a rich structural diversity. Varying the ligand structure makes it possible to change both the effective charge on the metal atom and the steric accessibility of this atom, which have a decisive effect on the catalytic properties of the complex.

In group 13, aluminum complexes have been most extensively studied as initiators for the ROP of cyclic ethers, and there are numerous reviews detailing the progress in this area over the past decade [43,111]. On the contrary, studies concerning heavy complexes of group 13 metals (for example, Ga, In) remain poorly studied, despite the well-known better stability of Ga particles (compared to Al) in a polar/proton environment [154]. This fact is of particular interest, given that for any ROP catalyst in industrial use, resistance to proton impurities is important.

In contrast to aluminum alkyl complexes, in some cases, it is impossible to obtain gallium alkyl derivatives directly through the interaction of trialkylgallium and a free ligand, which directly depends on the structure of the ligand. Thus, the authors of [155] found that the interaction of salen ligands with a stoichiometric amount of GaMe_3_, due to the low nucleophilicity of the methyl group, led under various reaction conditions to a complex mixture of difficult-to-identify compounds, which is consistent with the conclusions from review [156]. In such cases, it is possible to obtain methyl derivatives of gallium only through the formation of a lithium or sodium salt of the corresponding ligand and the interaction of this salt with gallium trihalide, leading to the target compound after the exchange reaction with MeLi. Gallium chloride complexes are also the starting point for the synthesis of other gallium derivatives (LGaX, X = Me, OAlk, CH_2_SiMe_3_).

Horegald and his collaborators [157] in 2010 described the first successful example of a gallium-based initiator capable of polymerizing cyclic esters. Previously, only an example of a gallium complex (BDI)GaClX (where X = Cl, OSO_2_CF_3_, OSitBu_3_; BDI = monoanionic β-diiminate ligand) was described, which was inactive in ring-opening polymerization [158].

**Scheme 13:**
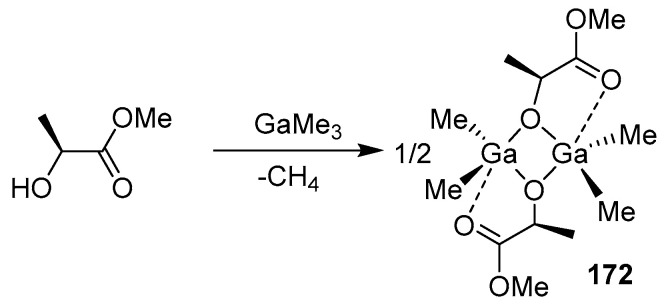
Synthesis of gallium complex **172**.

Compound **172** was obtained in an almost quantitative yield in an equimolar reaction between Me_3_Ga and (S)-methyl lactate (Scheme 13). Compound **172** effectively initiates *rac*-LA ROP to form narrowly dispersed atactic PLA (best experience: 62% conversion, [*rac*-LA]:[**172**] = 250:1, 40 °C, CH_2_Cl_2_, 48 h, *Đ* = 1.1). Notably, the addition of a Lewis base, such as γ-picoline, to compound **172** resulted in a stereoselective ROP process of *rac*-LA to produce heterotactically enriched PLA (P_r_, up to 78%), thus indicating that, in such systems, the coordination of the external base Lewis center Ga is clearly advantageous.

The addition of diazabicycloundecene (DBU) and 7-methyl-1,5,7-triazabicyclo [4.4.0]dec-5-ene (MTBD) bases [159] to compound **172** substantially weakens the Ga–O_alkoxide_–Ga bridge, which influences the stereoselectivity of the complex in the ring-opening polymerization of *rac*-LA, thereby obtaining a diblock polylactide consisting of isotactic and heterotactic enriched blocks.

**Scheme 14:**
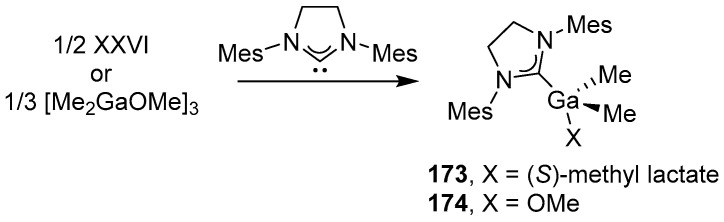
Synthesis of gallium complexes **173**–**174**.

In the course of the further development of this class of compounds, Ga(III) complexes were obtained, but using an N-heterocyclic carbene (in this case, SIMes) as an external Lewis base, which made it possible to obtain the corresponding alkoxide complexes (SIMes)Ga **173** and **174** (Scheme 14) [160].

Interestingly, compounds **173** and **174** showed significantly higher polymerization rates (compared to **172**) in *rac*-LA ROP and are claimed to give highly isotactic PLA (best experience with **173**: quantitative conversion, [*rac*-LA]:[**173**] = 50:1, −20 °C, CH_2_Cl_2_, 30 min, *Đ* = 1.11, P_m_ > 0.99). The improved activity of **173** and **174** in ROP was due to the increased basicity of the Ga-OR moiety, and also, in the case of the gallium lactate derivative **173**, the lack of C=O coordination, unlike compound **172**. Thus, in these systems with gallium lactate, the stereoselectivity switching from heterone to isoselectivity can be easily achieved by changing the nature of the external Lewis base (transition from γ-picoline to SIMes).

The tetracoordinated Ga(III) complex of the (NON)GaNMe_2_ **175** type, in which the dianionic ligand of N,O,N-diamidoether determines the trigonal-monopyramidal geometry of the gallium center, is highly reactive as an ROP initiator of cyclic ethers (*rac*-LA and CL) and trimethylene carbonate (TMC) [161].

The gallium complex **175** was obtained as a result of the elimination reaction of the amine Ga(NMe_2_)_3_ with the corresponding ligand.

Compound **175** initiates the controlled ROP of *rac*-LA under mild conditions to yield controlled-chain-length PLA (quantitative conversion, [*rac*-LA]:[**175**] = 50:1, 80 °C, in toluene solution, 1 h, M_n_ = 12,390 g/mol, *Đ* = 1.11). It is noteworthy that the activity in the ROP of the Ga **175** complex is higher than that of the Al complex on the same ligand. Also for **175**, the ROP of *rac*-LA was studied in the presence of a benzyl alcohol (BnOH) co-initiator, ε-CL, for 3 h at room temperature in CH_2_Cl_2_ (M_n_ = 12,507 g/mol, *Đ* = 1.05).

**Scheme 15:**
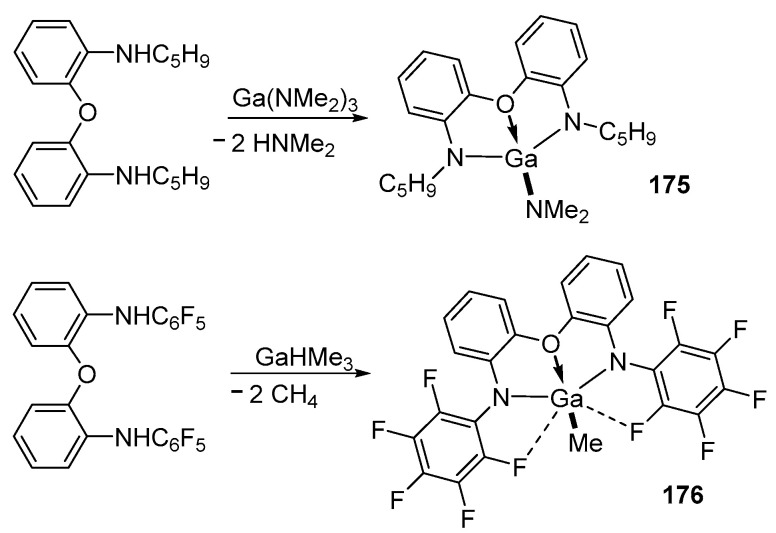
Synthesis of gallium complexes **175–176**.

The closely related gallium complex, **176**, is not active in the ROP process, most likely due to steric factors: the molecule contains two rather short Ga←F(o-C_6_F_5_) contacts, which prevents the monomer from approaching the gallium center [161,162] (Scheme 15).

**Scheme 16:**
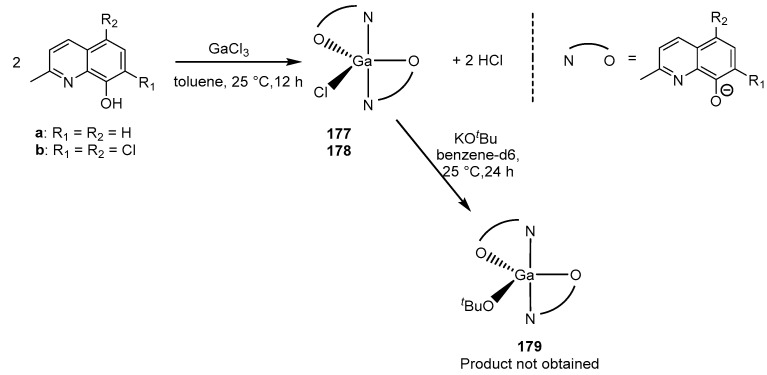
Synthesis of gallium complexes **177**–**179**.

The successful results and conclusions that gallium complex **175** had a higher reactivity in ROP compared to similar gallium complexes prompted the authors of [163] to synthesize and study the reactivity of Ga complexes **177** and **178** (Scheme 16). Previously, the authors reported on a series of bis(8-quinolinolate)aluminum ethyl complexes, which, in the presence of isopropyl alcohol, were effective isoselective initiators (P_m_ = 0.76) in *rac*-LA ROP [164]. Compounds **177** and **178** were tested as initiators of *rac*-LA ROP (1 M solution of LA in THF, 298 K, 20 h), but showed no activity. This is consistent with the conclusions of other researchers that metal halide complexes are rarely effective initiators, apparently due to the high strength of the M–Cl bond (481 kJ/mol versus 285 kJ/mol for Ga–O) [158].

For the synthesis of active initiators, gallium chloride complexes were subjected to further reaction with the formation of new alkoxide and amide complexes. Thus, attempts were made to carry out various salt metathesis reactions with the corresponding potassium salts (Scheme 15). The reaction between **178** and potassium *tert*-butoxide gives a yellow powder, the ^1^H NMR spectrum of which indicates the presence of the desired alkoxide complex **179** and a second complex containing only quinolinolate moieties, a single crystal of which the authors were able to isolate. The authors hypothesized that the oxygen/water required for such a decomposition reaction arises from residual oxygen/water in the solvents from the reaction or from the NMR spectrum. They also note that the same solvents were successfully used to isolate alkyl complexes of aluminum and gallium without any such decomposition, which indicates a high sensitivity of alkoxide derivatives. The pure alkoxide complex **179** was prepared via a reaction in an NMR tube with a Young’s tap and centrifugation to remove the KCl by-product.

**Scheme 17:**
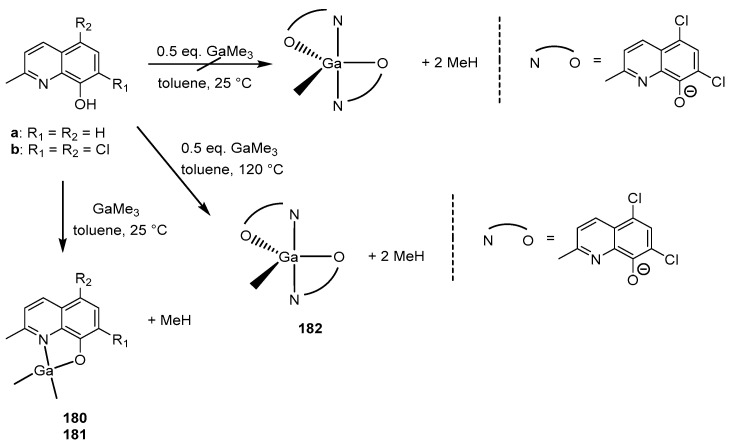
Synthesis of gallium complexes **180**–**182**.

As an alternative to gallium alkoxide complexes, gallium alkyl complexes [L_2_GaR] (R = alkyl) were considered as suitable precursors for the further protonolysis reaction with various alcohols. The reaction of two equivalents of ligands a and b with trimethylgallium resulted only in the formation of monoligand complexes **180** and **181** (Scheme 17). It is important to note that the reaction was carried out under the same conditions that previously made it possible to isolate aluminum ethyl bisligand complexes [L_2_AlEt] [164]. Changing the conditions, for example by using more polar solvents such as THF or pyridine, resulted exclusively in the formation of the same monoligand product. Complex **182** was obtained only through refluxing in a toluene solution in 25% yield.

Compounds **179**–**182** were studied as initiators in *rac*-LA ROP. The polymerization was carried out in toluene at 75 °C with the addition of an ^i^PrOH co-initiator. ([*rac*-LA] = 1 M, [I] = 0.01 M.) The activity of the initiators varied as follows: **181** > **180** > **182**. In the case of complex **179**, the polymerization was carried out in the absence of ^i^PrOH, and the monomer conversion reached 91% in 51 h; this rate was similar to the previously described analogous aluminum initiators [157,161,164]. The authors suggest that the increased activity of gallium compounds is due to the reduced Lewis acidity of Ga, which leads to a weaker or more labile gallium alkoxide bond. In addition, the Ga complex also exhibits high isoselectivity and good polymerization control (compared to the Al analog).

**Scheme 18:**
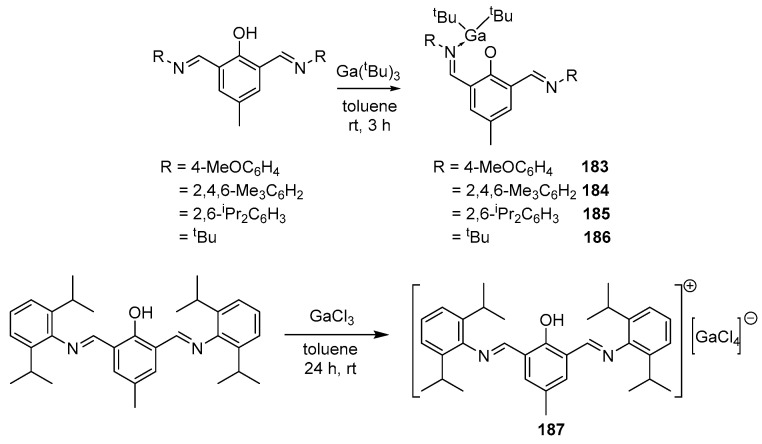
Synthesis of gallium complexes **183**–**187**.

The interaction of tri-*tert*-butylgallium with various bis(imino)phenolate ligands in a 1:1 stoichiometric ratio in dry toluene at room temperature led to the formation of heteroleptic complexes **183**–**186** (Scheme 18). Gallium complex **187** was obtained by adding an excess of GaCl_3_ to the corresponding ligand in dry toluene. Complexes **184** and **186** are monomeric in the solid phase. For complexes **183**–**186**, polymerization studies were carried out using *rac*-LA as a monomer (polymerization was carried out in bulk, at a temperature of 140 °C). During polymerization, the *Đ* of the polymers varied from 1.02 to 1.08, and a good agreement was also observed between M_theor_ and M_GPC_ at monomer loadings up to 2000. The catalytic activity decreased in the following order: **186** > **185** > **184** > **183**. These complexes during the polymerization of *rac*-LA lead to the formation of isotactic polylactide, due to the presence of two bulky groups directly associated with the metal center [147].

Aminophenols used as ligands for gallium complexes are very attractive due to their multifunctionality. OH and NH groups can react with alkyl gallium compounds to eliminate alkyl groups and form oxygen–metal and nitrogen–metal bonds. In addition, oxygen and nitrogen atoms are able to coordinate with metal centers. Aminophenolic units may also act as chelating ligands or bridge metal centers.

**Scheme 19:**
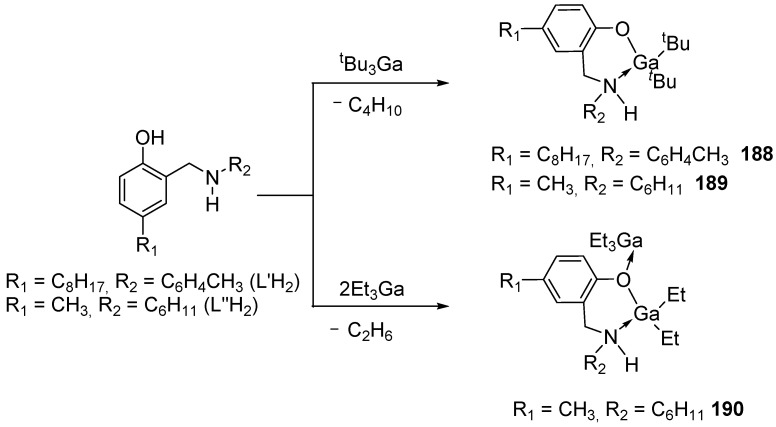
Synthesis of gallium complexes **188**–**190**.

The interaction of ^t^Bu_3_Ga with aminophenol ligands in a 1:1 molar ratio [81] gave monomeric gallium complexes: [^t^Bu_2_Ga(L’H)] (**188**) and [^t^Bu_2_Ga(L″H)] (**189**) (Scheme 19).

It should be noted that the replacement of the initial alkylgallium derivative ^t^Bu_3_Ga with R_3_Ga (R = Me, Et) leads to a mixture of difficult-to-identify compounds in the reactions of aminophenols L′H_2_ and L″H_2_ with R_3_Ga (R = Me, Et). The authors were able to isolate and characterize only one gallium ethyl complex, Et_2_Ga(L″H)Et_3_Ga (**190**) (Scheme 18). The catalytic activity for complexes **188**–**190** was tested in ε-CL ROP (110 °C with and without the addition of benzyl alcohol). According to the results, the catalytic activity of **190** is higher than that of **189**, and complex **188** showed a complete absence of catalytic activity. The resulting polymers are characterized by a narrow polydispersity index (*Đ* = 1.30).

**Scheme 20:**
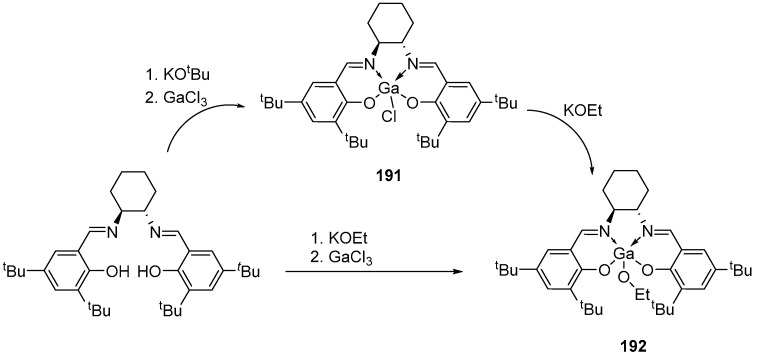
Synthesis of gallium complexes **191**–**192**.

In [165], gallium alkoxides based on diaminophenols and salen-type ligands were obtained for direct comparison with indium catalysts with similar ligands. Based on the salen-type ligands (±)-H_2_(ONNO), the authors obtained the chloride complex (ONNO)GaCl, (±)-**191** (Scheme 20). The further interaction of [ONNO]GaCl with one equivalent of KOEt leads to the formation of [ONNO]Ga(OEt) alkoxide complexes. Using an alternative method, the (±)-**192** complex can be obtained with a better overall yield by not isolating the intermediate gallium chloride complex, but by reacting directly to complete conversion to the desired complex **192** in the presence of excess KOEt.

**Scheme 21:**
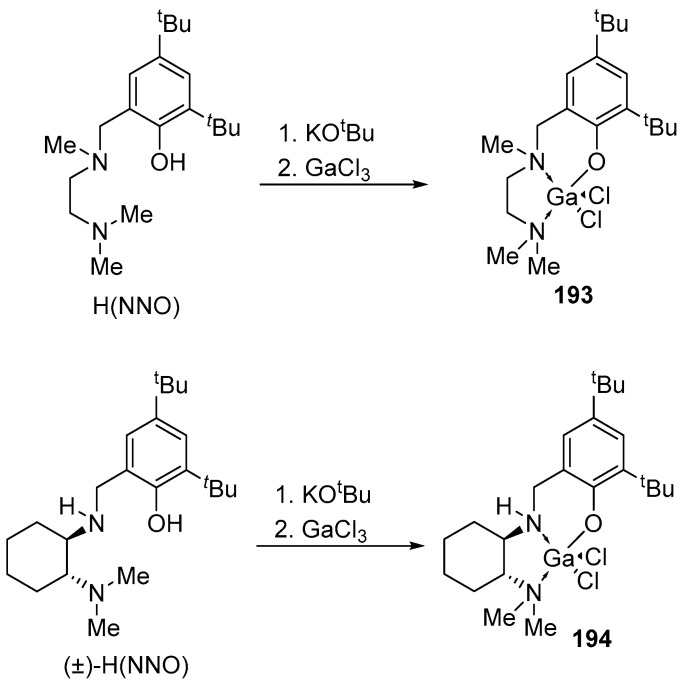
Synthesis of gallium complexes **193**–**194**.

Gallium dichloride complexes **193** and **194** were obtained as a result of the metathesis reaction between the dipotassium salt of the ligand K(NNO) and (±)-K(NNO) and GaCl_3_. Unlike their indium analogs [130,131,132,148,166], dichlorogallium complexes **193** and (±)-**194** do not react with KOEt at room temperature (Scheme 21). When potassium benzylate is added, complexes **193** and (±)-**194** form a mixture of substances, regardless of the conditions used; however, a single crystal of the target product was isolated from the reaction mixture (Scheme 20) [130,131,132,148,166].

The gallium (±)-**194** complex shows low activity in LA ROP (7 days, no co-initiator, toluene, 100 °C, *Đ* = 1.5, 81% conversion). Complex **194** turned out to be inactive for the polymerization of ε-caprolactone. Reactions in various solvents, as well as an increase in the polymerization temperature to 100 °C, did not lead to the production of polycaprolactone.

**Scheme 22:**
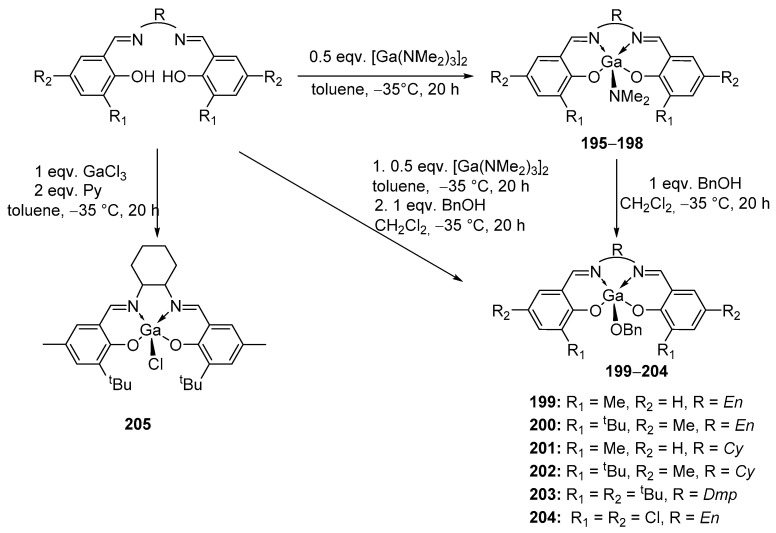
Synthesis of gallium complexes **195**–**205**.

The reaction of corresponding ligands with a stoichiometric amount of GaMe_3_ in all cases led to a complex mixture of compounds under various reaction conditions. Isolation of the amine, in the case of [Ga(NMe_2_)_3_]_2_ (compared to GaMe_3_) as a Ga(III) precursor, allowed for the direct synthesis of the corresponding (salen) Ga–NMe_2_ complexes at room temperature (**195**–**198**; Scheme 22).

(Salen)gallium benzyl oxide complexes **199**–**204** [155] were prepared by reacting BnOH with the corresponding (salen)gallium amide complexes (**195**–**198**) (Scheme 21). The authors also evaluated the activity of gallium complexes **198** and **199**–**203** with respect to *rac*-LA and ε-CL. Complexes **199**–**201**, **203** and **198** initiated controlled the ROP of *rac*-LA (toluene, 90 °C, conversion from 40 to 95%) to obtain isotactically enriched PLA (Pm = 0.68–0.77) and narrow MWD (1.10–1.16). In contrast, the ROP of lactide initiated by **202** resulted in heterotactically enriched PLA (P_m_ = 0.38). The activity of Ga complexes varies in the following order: **198**/BnOH ≈ **199** > **203**/BnOH > **201** > **202** > **200**. The complex **199**, as in the case of *rac*-LA, also easily initiates the polymerization of ε-CL ([ε-CL]_0_ = 1 M, 2 h, toluene, 90 °C, 93% conversion) to obtain narrowly dispersed PCL (*Đ* = 1.19).

**Figure 29:**
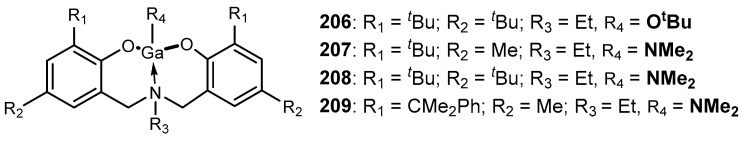
Gallium complexes **206**–**209**.

The authors of [167] reported on the synthesis of the first examples of gallium complexes based on “privileged” aminobisphenolate ligands (Figure 29). These complexes turned out to be extremely active in the ring-opening polymerization of ε-caprolactone, even at room temperature, and were highly active in the ROP of L-LA.

Gallium complexes **206**–**209** were tested as catalysts for the ROP of ε-CL, L-LA and their copolymerization. The polymerization of ε-CL was carried out in bulk at 80 °C. Compound **208** was also tested as a ε-CL ROP catalyst at 25 °C and 100 °C; all polymerizations of L-LA were also carried out in the absence of a solvent at 100 °C. Complex **208** is extremely active with regard to the controlled polymerization of ε-caprolactone, as evidenced by the conversion of monomers and the relatively narrow polydispersity of PCL (*Đ* = 1.38). It should be noted that the activity of initiator **208** is the highest (100% conversion, 15 min, 25 °C) among the gallium complexes that have been previously studied as initiators of caprolactone polymerization. An analysis of the end groups of isolated PCL-200 from the ^1^H spectrum shows that the PCL chain is closed by one dimethylamide and one hydroxyl end of the chain. This fact confirms that, in this case, the “coordination−insertion” ROP mechanism is realized with the transfer of the amino group from the metal atom to the end of the polymer chain. The M_n_ values obtained from the PCL samples were below the expected values based on the % of conversion. This deviation probably means that transesterification side reactions and/or a slow initiation step have taken place. To demonstrate the feasibility of synthesizing PCL with a high M_n_ content and well-controlled nature, the authors performed the ROP of ε-CL with [ε-CL]/[**208**] = 500 at 100 °C for 30 min. All synthesized complexes were also tested in L-LA ROP and showed slightly lower activity compared to ε-CL. The authors chose gallium alkoxide complex **206** as an initiator for the copolymerization of L-LA and ε-CL, since this complex in ROP shows similar activity for ε-CL and L-LA. During the copolymerization of ε-CL and L-LA, the homopolymerization of ε-CL proceeded faster compared to L-LA, which is consistent with the calculations performed using the density functional theory method [168].

## 5. Metal Complexes as Initiators for Controlled Copolymerization of Lactide with Lactones and Cyclic Carbonates 

PLA is currently still an attractive and useful class of biodegradable polyesters among the numerous polyesters that have been studied to date. However, PLA has a number of disadvantages, such as poor elasticity and gas/water permeability, as well as brittleness and low thermal stability, which limit its range of potential applications [169].

One way to overcome this problem involves the ring-opening copolymerization of LA with other comonomers such as PCL, polyhydroxybutyrate (PHB) and modified cyclic carbonates [170], resulting in a polymer with repeating units of chemically different, covalently linked monomers. In this way, it is possible to solve the problems of poor interfacial adhesion or immiscibility that may arise during polymer plasticization [171]. Thus, the copolymerization of LA in the presence of caprolactone allows the properties of each homopolymer to be included in the resulting copolymer. The two homopolymers have opposite physical and thermal properties, making them complementary. For example, polycaprolactone (T_g_(PCL) = −60 °C) has good permeability and elasticity, but poor mechanical properties, namely viscosity [172], which is the opposite of the characteristics of PLA (T_g_(PLA) ≈ 57 °C). Fine-tuning the properties of a copolymer can be achieved by changing its microstructure (stereoregularity, comonomer distribution) as well as its macromolecular values (molecular weight, molecular weight distribution). Thus, biodegradable materials with improved properties can be obtained by copolymerizing LA with lactones. However, the difference in the reactivity ratios of LA and ε-CL makes it difficult to prepare a random copolymer. Thus, the development of effective catalysts for the statistical copolymerization of LA and ε-CL is an urgent task. A recent review of metal-based catalytic systems for the controlled random copolymerization of lactide with lactone presented various metal-based catalysts and/or initiators for the synthesis of random copolymers of LA and lactone under smooth conditions, allowing for the best possible controlled polymerization process [21].

As for CL/LA, block and gradient copolymers are usually formed during BBL/LA copolymerization due to the slower rate of incorporation of β-butyrolactone into the polymer compared to lactide, while random copolyesters are rarely obtained [173]. Interestingly, effective catalysts for the copolymerization of LA and BBL remain scarce, despite the fact that various metal complexes, such as group 4 and group 13, are described as active catalysts, giving block copolymers [174,175,176] and gradient copolymers [124,177,178,179].

Also, one of the options for improving the properties of PLA is the copolymerization of LA with 1,3-trimethylene carbonate (TMC). PTMC is a class of biocompatible and flexible scaffold materials; more importantly, PTMC does not produce acidic products during the decomposition process. The use of PTMC and related copolymers in tissue engineering has been widely studied due to its easily tuned properties [180,181]. The copolymerization of LA with TMC can provide copolymers with characteristics that combine soft polyesters with relatively low T_g_. However, the random copolymerization of LA with 1,3-trimethylene carbonate (TMC) is still a challenge. Some calcium complexes based on Schiff [182] and 2,2-dibutyl-2 stanna1,3-dioxepane (DSDOP) [183] have been developed for TMC/LA copolymerization; however, due to the fact that LA is consumed much faster than TMC during copolymerization, these copolymerization reactions always result in the formation of blocky/trapezoidal microstructures with relatively long PLA blocks. It should be noted that, to date, it has been reported that only a tin-based catalyst is capable of promoting the copolymerization of L-LA and TMC to produce random copolymers via random copolymerization (r_LA_ = 1.03; r_TMC_ = 0.76); however, copolymerization should be carried out at high temperatures (180 °C) [184,185]. Therefore, the development of effective LA/TMC random copolymerization initiators under mild conditions is highly desirable.

**Figure 30:**
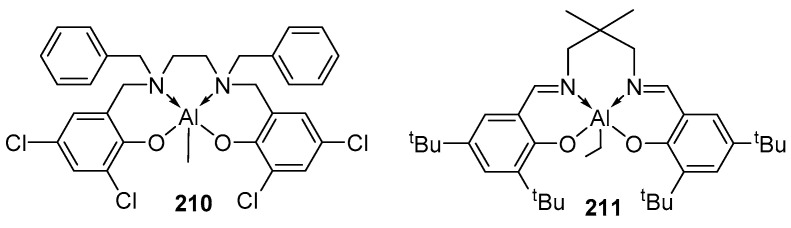
Aluminum complexes **210**–**211**.

The authors of [186] studied complexes **210** (L_1_AlMe, L_1_ = N,N′-dibenzyl-N,N′-bis[(3,5-dichloro-2-hydroxyphenyl)methylene]-1,2-diaminoethane) and **211** (L_2_AlEt, L_2_ = N,N′-(2,2-dimethylpropylene)-bis(3,5-ditert-butylsalicylideneimine) (Figure 30) in the copolymerization of LA (L-LA or *rac*-LA) with a functionalized cyclic carbonate (MAC), which previously showed excellent stereoselectivity in *rac*-LA ROP (for **210** P_r_ = 0.96, for **211** P_m_ = 0.90 [187]). It was found that the homopolymerization rate constant in the presence of complex **210** L-LA, *rac*-LA or MAC follows the trend k_app_ (MAC) > k_app_ (rac-LA) > k_app_ (L-LA), which indicates that complex **210** is most active in the case of MAC homopolymerization compared to LA monomers. Coordinating ability of L-LA than that of MAC, which interrupts the rapid homopolymerization of MAC, and the high heteroselectivity of complex **210** makes it difficult to sequentially grow L-LA, while the homopolymerization of MAC is slowed down due to its weaker coordinating ability. Thus, the cross-polymerization of L-LA and MAC is favorable (kML ≈ kLM, r_L-LA_ = 0.217 and r_MAC_ = 0.578), forming a random poly(MAC-r-L-LA) structure. In the case of the copolymerization of *rac*-LA and MAC, there is a trend towards the homopolymerization of MAC rather than cross-polymerization with *rac*-LA (r_MAC_ = 2.478 and r_rac-LA_ = 0.584). As a result, a gradient microstructure of poly(MAC-grad-rac-LA) was obtained. During the copolymerization of L-LA and MAC in the presence of complex **211**, L-LA was introduced into the copolymer much faster than MAC (k_LL_ ≫ k_LM_), which is explained by the different selectivity of the two systems: heteroselective **210** prefers the alternating polymerization of L-LA and D-LA, and isoselective **211** prefers the sequential incorporation of the same enantiomer. The monomer reactivity ratios of r_L-LA_ = 2.035 and r_MAC_ = 0.112 (rL-LA ≫ rMAC) indicate that L-LA also tends to homopolymerize rather than copolymerize with MAC. For the copolymerization of rac-LA and MAC, catalyzed by complex **211**, the reactivity ratios of the monomers r_rac_-LA = 1.619 and rMAC = 0.283 were obtained.

**Figure 31:**
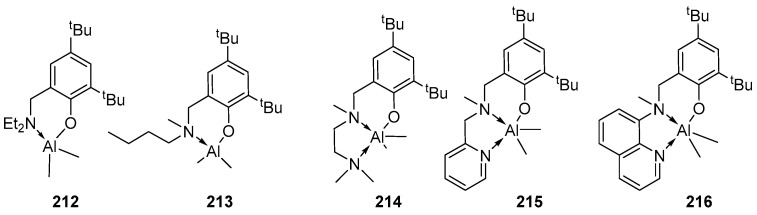
Aluminum complexes **212**–**216**.

In [188], a series of aluminum complexes based on aminophenol-type ligands were prepared, which were studied in the living ROP of lactones, such as ε-CL and δ-valerolactone (δ-VL), and their block copolymer in the presence of BnOH at room temperature (Figure 31). The resulting polymers had a high molecular weight (M_w_) up to 303 kg·mol^−1^ and a narrow molecular weight distribution (*Đ*~1.12). It should be noted that changing the ligand structure, namely varying the side arm, plays an important role in the reactivity of these catalytic systems. In particular, the presence of a butyl substituent in the side branch in complex **213** led to an increase in polymerization activity, and the presence of a bulkier side branch in complex **216** led to a decrease in its activity. It is also worth noting that the presence of pyridine in the side branch in complex **215** leads to the effective inhibition of transesterification processes.

PVL and PCL, as two types of important aliphatic polyesters, have almost similar properties in chemistry and biological medicine, which greatly limits its application in various fields. However, due to its good biocompatibility, biodegradability and permeability, PVL can be used as a hydrophobic block in amphiphilic block copolymers for in vivo chemotherapeutic drug delivery systems [189,190]. On the other hand, the high crystallinity and hydrophobicity allow PCL to generally show a slow degradation rate (1–2 years), so its use is limited to a long lifespan [172]. Copolymerization provides an effective strategy for easily tailoring material properties by adjusting the copolymer composition to synthesize polymers with different properties. For example, by adding a second comonomer such as L-lactide, δ-VL, γ-butyrolactone (γBL), γ-valerolactone (γ -VL) or γ-caprolactone (γ-CL) randomly observed in PCL chains accelerate the rate of decomposition. 

**Figure 32:**
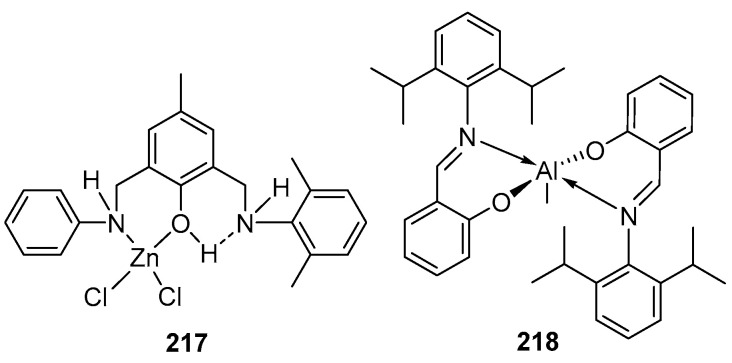
Zinc complex **217** and aluminum complex **218**.

The authors of [191] reported on the use of zinc complexes containing 2,6-bis(amino)phenolic ligands as a catalyst for δ-VL ROP and copolymerization with ε-CL. In combination with four eq. zinc methyllithium complexes containing 2,6-bis(amino)phenol, (Figure 32) the ligands showed very high activity against ε-CL ROP, both in the presence and in the absence of BnOH [128]. At the same time, the zinc complex **217**/MeLi catalytic system in δ-VL ROP also showed high activity in the presence or absence of BnOH, with the complete conversion of δ-VL in 3–10 min. PVL obtained in the presence of BnOH has a lower molecular weight ([VL]_0_/[**217**]/[BnOH]: 200/1/0, M_n_ = 13,100, *Đ* = 1.62; [VL]_0_/[**217**]/[BnOH ]: 200/1/1, M_n_ = 10,300, *Đ* = 1.25) and narrower molecular weight distribution than PVL prepared without BnOH. Due to the good ROP characteristics of ε-CL and δ-VL, the 1/MeLi zinc complex system was additionally used in the copolymerization of ε-CL and δ-VL to produce random and block copolymers, using various monomer feeding regimes. Random copolymers [P(CL-co-VL)] were prepared by premixing the two monomers, and block copolymers (PCL-b-PVL and PVL-b-PCL) were prepared in a sequential feed mode. The composition of the copolymer was controlled by changing the feed ratio of the two monomers. Due to the high activity, in most cases, total yields of 100% were achieved, and the content of VL/CL in the copolymers was close to the corresponding values in the starting material. With an equal ratio of ε-CL and δ-VL, the average sequence length is comparable, which indicates the formation of a random copolymer ([CL]_0_/[VL]_0_/[BnOH]: 150/150/0: *L*_c_ = 2.13; *L*_v_ = 2.49).

In [192], the aluminum complex [AlMeL_2_] (**218**) (Figure 32) turned out to be a universal catalyst for both the homopolymerization and copolymerization of cyclic ethers. The polymerization of *rac*-LA under the action of the **218**/BnOH catalytic system proceeded in a controlled manner (*Đ* < 1.10), and the resulting polylactide was heterotactically enriched (P_r_ = 0.69). Complex **218** was also effective in the copolymerization of L-LA and *rac*-β-butyrolactone. NMR data revealed that both L-LA and BBL monomers were included in the polymer chain, and the actual composition of LLA and BBL in the copolymer was similar to the original monomer loading. Also, the formation of a random copolymer of LLA and BBL is supported by the same glass transition temperature. When copolymerizing L-LA and ε-CL in a different range of compositions, complex **218** was also effective. In all cases, the amount of each comonomer in the final polymer was essentially the same as in the original monomer loadings. An analysis of copolymers of L-LA and ε-CL carried out using ^13^C NMR, as well as an analysis of thermal properties (only one glass transition temperature point was found for the resulting copolymers), showed that the resulting polymers are statistically relevant.

## 6. Conclusions and Perspectives

In general, alkoxide complexes of group 1 and 2 metals exhibit the highest activity, which is due to the polarity of the M–OR bonds. Complexes of group 12 and 13 metals, along with having a lower activity compared to group 1 and 2 metals, have excellent stereoselectivity in ROP. It is also worth noting that group 4 metal complexes, namely titanium, zirconium and hafnium, due to their high stability under reaction conditions, are also promising for industrial use, since their activity still exceeds the activity of tin octanoate.

Thus, ring-opening polymerization in the presence of metal-complex initiators continues to be the most effective method for the synthesis of cyclic ester polymers. The main directions of research were related to the search for new, more active and less toxic initiators. The most promising at the moment are zinc complexes, due to their activity and biocompatibility, which are the subject of a significant part of this review. Without denying the importance of searching for new initiators that are more active than existing ones, and at the same time maintaining stability in polymerization under industrial conditions, in our opinion, a promising area of research at the moment is the search for initiators for the synthesis of copolymers with different structures. Unfortunately, an analysis of data from the literature currently indicates a number of problems in the synthesis of copolymers that arise due to the different rates of polymerization of monomers. It is worth noting that the polymerization rates in the synthesis of homopolymers and in the synthesis of copolymers for the same monomers are very different. Therefore, the synthesis of new initiators and their study in the copolymerization of lactide with lactones is extremely important for the development of this branch of modern materials science.
